# Supplementary material for: Factors affecting mental health of health care workers during coronavirus disease outbreaks (SARS, MERS & COVID-19): A rapid systematic review
Source: PLoS One. 2020 Dec 15;15(12):e0244052. doi: 10.1371/journal.pone.0244052 (PMC7737991; doi:10.1371/journal.pone.0244052)
Supplement: S2 Table — (DOCX) [file pone.0244052.s002.docx]

**S3 Table. Detailed study characteristics**

| **Author**  **Publication year** | **Country** | **Epidemic** | **Study design** | **Participants** | **Intervention/risk factors/exposure** | **Outcome measures + measurement tool/scale** |
| --- | --- | --- | --- | --- | --- | --- |
| Bai 2004 | Taiwan | SARS | Cross-sectional study | 338 staff members including HCWs, and administrative personnel | - Quarantine | - Acute stress/post-traumatic stress (symptoms): Acute stress disorder, according to DSM-IV |
| Chan 2004 | Singapore | SARS | Cross-sectional study | 661 HCWs including doctors and nurses | - Clear communication of directives - Being able to give feedback to management - Support from supervisors and colleagues - Support from family - Being able to talk someone - Exposure to SARS - Religious convictions | - Acute stress/post-traumatic stress (symptoms): Post-traumatic stress, Impact of Event Scale - General symptoms of psychopathology: Psychiatric symptoms, 28-item General Health Questionnaire |
| Chang 2006 | Taiwan | SARS | Cross-sectional study | 211 HCWs including doctors and nurses | - Social interaction with colleagues - Trust (to be able to rely on colleagues) | - Emotional exhaustion and burnout: Emotional exhaustion, measured with a questionnaire developed by the researcher |
| Chen 2005 | Taiwan | SARS | Cross-sectional study | 128 nurses | - Working in a high risk unit (vs working in low risk unit) - Being conscripted from a lower risk unit to a higher risk unit (vs working in low risk unit) | - Acute stress/post-traumatic stress (symptoms): Post-traumatic stress, Impact of Event Scale - Anxiety-related symptoms: 90-item Symptom Checklist-Revised - Depression-related symptoms: 90-item Symptom Checklist-Revised |
| Chen 2006 | Taiwan | SARS | Uncontrolled before-and-after study | 116 volunteers from the nursing staff | - SARS prevention program   The program included a series of in-service training, detailed manpower allocation, adequate protective equipment, and the availability of a mental health team. | - Anxiety-related symptoms: Zung’s self-rating anxiety scale - Depression-related symptoms: Zung’s self-rating depression scale - Sleep problems: Sleep quality, Pittsburgh sleep quality index |
| Chen 2007 | Taiwan | SARS | Cross-sectional study | 90 HCWs including physicians, critical care nurses, respiratory care specialists and others  and 82 control subjects including hospital administrators and employees who  had no history of contact with SARS patients | - Exposure to SARS patients (as defined by HCW and controls subjects) | - General symptoms of psychopathology: Mental health, part of the general health status, as measured by the Medical Outcome Study Short-Form 36 Survey |
| Chong 2004 | Taiwan | SARS | Cross-sectional study | 1257 staff members including nurses, doctors and health administrative workers | - Exposure to SARS - Care for SARS patients | - General symptoms of psychopathology: Psychiatric morbidity, 12 item Chinese Health Questionnaire (use of cut-off score) |
| Ho 2005 | Hong Kong | SARS | Cross-sectional study | 97 HCWs who had been infected including doctors, nurses, allied health professionals and support staff | - Fear related to SARS: SARS Fear Scale: - Infection: fear of infecting either themselves or close family members - Insecurity: fear of potential harm, death, and life out of control - Instability: work–environment changes, including fear of isolation, a heavy workload, and assignment to the SARS ward. - Perceived self-efficacy: Chinese Self-Efficacy Scale | - Acute stress/post-traumatic stress (symptoms): Post-traumatic stress symptoms, The Chinese Impact of Event Scale – Revised |
| Kang 2020 | China | COVID-19 | Cross-sectional study | 994 HCW (medical and nursing staff) | - Exposure to COVID-19 (contact with patients or self/friend/family infected) - Accessed mental health care services (received psychological materials in the hospital, psychological assistance methods available via online media and platforms) | - General symptoms of psychopathology: “mental health”, based on a cluster analysis according to the scores of the following outcome measures: - Acute stress/post-traumatic stress (symptoms): 22-item Impact of Event Scale - Anxiety-related symptoms: 7-item Generalized Anxiety Disorder - Depression-related symptoms: 9-item Patient Health Questionnaire - Sleep problems: Insomnia symptoms, Insomnia Severity Index |
| Kim 2016 | South Korea | MERS | Cross-sectional study | 215 nurses working in the emergency departments | - MERS-related job stress - Fear of MERS-infection - Support from family and friends | - Emotional exhaustion and burnout: MERS-related burnout, Oldenburg Burnout Inventory |
| Koh 2005 | Singapore | SARS | Cross-sectional study | 10511 staff members from 3 SARS and 6 SARS-free hospitals, including all employees on the payroll | - Health care institution: non-SARS affected vs SARS-affected hospital - Extent of exposure to SARS: rarely exposed vs daily exposure | - (Perceived) stress: stress at work, questionnaire developed by researchers |
| Lai 2020 | China | COVID-19 | Cross-sectional study | 1257 HCWs including physicians, and nurses | - Working position: frontline vs second-line   (Frontline HCW were directly engaged in diagnosing, treating, or providing nursing care to patients with elevated temperature or patients with confirmed COVID-19) | - Depression-related symptoms: 9-item Patient Health Questionnaire - Anxiety-related symptoms: 7-item Generalized Anxiety Disorder - Sleep problems: Insomnia symptoms, Insomnia Severity Index - Acute stress/post-traumatic stress (symptoms): Distress symptoms, 22-item Impact of Event |
| Lancee 2008 | Canada | SARS | Cross-sectional | 587 HCWs including  nurses in medical or surgical inpatient  units and all staff of intensive care units, emergency departments, and SARS isolation units | - Perception of adequate training and support | - General symptoms of psychopathology: Psychological disorder, according to DSM-IV |
| Liu 2012 | China | SARS | Cross-sectional study | 549 HCWs including doctors and nurses | - Working in a high risk location - Any quarantining - Perception of SARS-related risks - Job stress - Altruistic acceptance | - Depression-related symptoms: Center for Epidemiologic Studies Depression Scale |
| Lu 2020 | China | COVID-19 | Cross-sectional study | 2299 HCWs including 2042 medical staff and 257 administrative staff | - High risk contact (vs no contact, i.e. non-clinical staff) | - Anxiety-related symptoms: level of fear measured with numeric rating scale - Anxiety-related symptoms: Hamilton Anxiety Scale - Depression-related symptoms: Hamilton Depression Scale |
| Marjanovic 2007 | Canada | SARS | Cross-sectional study | 333 nurses | - Levels of vigor - Organizational support - Trust in equipment/infection control initiative - Contact with SARS patients - Time spent in quarantine | - Emotional exhaustion and burnout: Emotional exhaustion, Maslach Burnout Inventory-General Survey - Anger: State anger, State-Trait Anger Expression Inventory |
| Maunder 2004 | Canada | SARS | Cross-sectional study | 1557 HCWs including doctors, nurses, laboratory staff, and others | - Job stress - Social isolation and avoidance - Health fear - Doubt about protection - Dissatisfaction with hospital system and procedures - Contact with patients with SARS | - Acute stress/post-traumatic stress (symptoms): Psychological stress, Impact of Event scale |
| Maunder 2006 | Canada | SARS | Cross-sectional study | 769 HCWs including physcians, nurses, and others | - Maladaptive coping - Perceived adequacy of training, protection and support - Stigma and avoidance - Adaptive coping - Worked on SARS unit - Ever in SARS patient room - Touched SARS patient - Being quarantined - Unprotected exposure | - Acute stress/post-traumatic stress (symptoms): Post-traumatic stress, Impact of Event Scale - General symptoms of psychopathology: Psychological stress, Kessler Psychological Distress Scale - Emotional exhaustion and burnout: Professional burnout, Emotional Exhaustion Scale of the Maslach Burnout Inventory |
| McAlonan 2007 | Hong Kong | SARS | Cross-sectional study | 106 high-risk HCWs who practiced respiratory medicine (high risk group) and 71 control subjects including non-respiratory medicine workers | - Risk of exposure to SARS: high risk vs low risk (as defined by high-risk and control subjects) | - (Perceived) stress: Perceived stress scale - Depression-related symptoms: subscale of Depression and Anxiety Scale - Anxiety-related symptoms: subscale of Depression and Anxiety Scale - (Perceived) stress: subscale of Depression and Anxiety Scale - Acute stress/post-traumatic stress (symptoms): Post-traumatic stress, Impact of Event Scale |
| Mo 2020 | China | COVID-19 | Cross-sectional study | 180 nurses | - Working hours per week | - (Perceived) stress: Chinese version of Stress Overload Scale |
| Nickell 2004 | Canada | SARS | Cross-sectional study | 2001 hospital employees including health care professionals, nurses and doctors. (only a subset, 510 employees, received the General Health Questionnaire) | - Being treated differently because of working in hospital - Perceived death rate of SARS - Precautionary measures sufficient - Precautionary measures affect ability to do job | - General symptoms of psychopathology: Emotional distress, 12-item General Health Questionnaire - Anxiety-related symptoms: Concern for personal of family’s health, based on closed and open-ended questions about the respondent’s concerns about SARS |
| Park 2018 | South Korea | MERS | Cross-sectional study | 187 nurses working in a high risk area | - Hardiness (measured with Dispositional Resilience Scale-15) - Perceived Stigma (measured with a newly developed scale) | - General symptoms of psychopathology: Mental Health, Mental Component Summary of Short Form-36 instrument - (Perceived) stress: Perceived stress scale-10 |
| Sim 2004 | Singapore | SARS | Cross-sectional study | 277 HCWs including doctors and nurses | - Coping strategy: self-distraction, instrumental support seeking, behavioural disengagement, venting, humor, acceptance, religion, active coping, denial, emotional support seeking, positive refraining, planning (measured by the Brief Cope questionnaire) - Work at fever room/tent - Contact with suspected SARS patient | - General symptoms of psychopathology: Psychiatric morbidity, 28-item General Health Questionnaire (use of cut-off score) - Acute stress/post-traumatic stress (symptoms): Post-traumatic morbidity, Impact of Event Scale |
| Son 2019 | South Korea | MERS | Cross-sectional study | 280 hospital workers including doctors, nurses, clinical technical/support staff and office workers.  A comparison between HCW and non-HCW is made | - Negative emotional experience - Perceived risk - Coping ability | - Acute stress/post-traumatic stress (symptoms): Likelihood of PTSD, Korean version of Impact of Event Scale - General symptoms of psychopathology: Negative emotional experience (“What emotion did you experience the most during the MERS outbreak”, 9-point Likert scale) |
| Styra 2008 | Canada | SARS | Cross-sectional study | 248 HCWs from high-risk units consisting of a  special SARS unit, the intensive care unit, and the emergency department | - Perception of risk to others - Confidence in infection control measures - Confidence in the information provided - Working in a high-risk unit - Perception of personal risk - Impact on work life | - Acute stress/post-traumatic stress (symptoms): Post-traumatic stress, Impact of Event Scale |
| Su 2007 | Taiwan | SARS | Cross-sectional study | 70 nurses from SARS units and 32 nurses from non-SARS units | - Working in SARS unit - Perceived negative feelings towards SARS - Positive attitude towards SARS | - Depression-related symptoms: Beck Depression Inventory - Acute stress/post-traumatic stress (symptoms): Post-traumatic stress symptoms, Chinese version of Davidson Trauma Scale - Sleep problems: Presence of insomnia, DSM-IV |
| Tam 2004 | Hong Kong | SARS | Cross-sectional study | 652 frontline HCWs including doctors, nurses, health care  assistants, physiotherapists and occupational  therapists | - Adequate insurance and compensation - Adequate counseling and psychological support from employer - Direct contact with SARS patients - Frontline staff feedback reaching administrators - Clear infection control guidelines - Expressing opinions through staff unions or mass media - Protective facilities and temporary residential arrangements - Sense of coherence and team spirit - Appreciation from the community - Support from relatives - Job-related stress | - General symptoms of psychopathology: Psychiatric morbidity, 12-item Chinese Health Questionnaire (with use of cut-off score) |
| Wong 2005 | Hong Kong | SARS | Cross-sectional study | 466 HCWs in the Emergency department including doctors, nurses and health care assistants | - Vulnerability/loss of control - Health of self - Spread of virus - Health of family/others - Changes in work (transferred to another ward) - Being isolated (being discriminated/alienated) | - General symptoms of psychopathology: Mental distress, newly designed scale |
| Wong 2007 | Canada | SARS | Cross-sectional study | 137 and 51 doctors from Hong Kong and Toronto respectively | - Formal training in handling infectious diseases in primary care | - Anxiety-related symptoms: Visual Analogue Scale |
| Wu 2009 | China | SARS | Cross-sectional study | 549 hospital employees including doctors, and nurses administrative or other hospital staff | - Being quarantined - Working in high-risk locations - Perceived risk level - Altruistic acceptance | - Acute stress/post-traumatic stress (symptoms): Post-traumatic Stress, Impact of Event Scale |
| Xiao 2020 | China | COVID-19 | Cross-sectional study | 180 medical staff in departments of respiratory medicine, fever clinics, or the intensive care unit including doctors or nurses | - Social support (measured by the Social Support Rate Scale) | - Anxiety-related symptoms: Self-Rating Anxiety Scale - (Perceived) stress: Self-efficacy, General Self-Efficacy Scale - (Perceived) stress: Stanford Acute Stress Reaction Questionnaire - Sleep problems: Sleep quality, Pittsburgh Sleep Quality Index |
| Zhang 2020 | China | COVID-19 | Cross-sectional study | 1563 medical staff members including frontline workers | - Contact with COVID-19 infected patients - Worried about being infected - Received sufficient infection prevention training - Current protection can prevent getting infected - Uncertainty regarding effective disease control | - Sleep problems: Insomnia symptoms, Insomnia Severity Index |
| Zhu 2020 | China | COVID-19 | Cross-sectional study | 165 HCW (79 doctors, 86 nurses) | - Coping style/positive coping (measured with Simplified Coping Style Questionnaire) | - Anxiety-related symptoms: self-rating anxiety scale - Depression-related symptoms: self-rating depression scale |

HCW: health care worker
