# Supplementary material for: Factors affecting mental health of health care workers during coronavirus disease outbreaks (SARS, MERS & COVID-19): A rapid systematic review
Source: PLoS One. 2020 Dec 15;15(12):e0244052. doi: 10.1371/journal.pone.0244052 (PMC7737991; doi:10.1371/journal.pone.0244052)
Supplement: S3 Table — (DOCX) [file pone.0244052.s003.docx]

**S4 Table. Summary of findings**

| **Outcome** | **Comparison/Risk factor/Exposure** | **Effect Size** | **Number of participants** | **Reference** |
| --- | --- | --- | --- | --- |
| **RISK FACTORS** | | | | |
| **Level of disease exposure** | | | | |
| **Acute stress disorder/post-traumatic stress (symptoms)** | | | | |
| Post-traumatic stress | Direct contact with SARS patients vs no direct contact with SARS patients | Not statistically significant:  aOR: 0.92, 95% CI [0.53;1.61]  (p=0.77) | 661 | Chan 2004 |
| Psychological stress | Contact with SARS patients | Not statistically significant:  β: 0.002, 95%CI [-0.054;0.059]*  (p=0.95) | 1557 | Maunder 2004 |
| Post-traumatic morbidity | Contact with suspected SARS patient | Not statistically significant:  aOR: 0.36, 95%CI [0.04;3.03]  (p=0.35) | 277 | Sim 2004 |
| Post-traumatic stress | Touched SARS patient | Not statistically significant (not entered in stepwise regression) | 587 | Maunder 2006 |
| Post-traumatic stress | Worked in SARS unit | Not statistically significant (not entered in stepwise regression) | 587 | Maunder 2006 |
| Post-traumatic stress | Ever in SARS patient room | Not statistically significant (not entered in stepwise regression) | 587 | Maunder 2006 |
| Post-traumatic stress | Working in a high risk unit (vs working in a low risk unit) | Not statistically significant:  19.1±14.2 vs 13.7±9.1  MD: 5.4, 95%CI [1.0;9.8]*  (p>0.05) | 65 vs 42 | Chen 2005 |
| Post-traumatic stress | High risk vs low risk exposure | Statistically significant:  β: 0.31, 95%CI [0.16;0.46]*  (p<0.001) | 184 | McAlonan 2007 |
| Post-traumatic morbidity | Work at fever room/tent | Not statistically significant:  aOR: 1.02, 95%CI [0.28;3.68]  (p=0.98) | 277 | Sim 2004 |
| Post-traumatic stress | Working in a high-risk unit | Statistically significant:  aOR: 2.2, 95%CI [1.0;4.4]  (p=0.04) | 248 | Styra 2008 |
| Post-traumatic stress | Working in high-risk locations | Statistically significant:  aOR: 2.09, 95%CI [1.03;4.26]  (p<0.05) | 549 | Wu 2009 |
| Distress symptoms | Frontline vs second-line | Statistically significant:  220/522 vs 220/735  aOR: 1.60, 95%CI [1.25;2.04]  (p<0.001) | 522 vs 735 | Lai 2020 |
| **Anxiety-related symptoms** | | | | |
| Anxiety symptoms | Frontline vs second-line | Statistically significant:  181/522 vs 184/735  aOR: 1.57, 95%CI [1.22;2.02]  (p<0.001) | 522 vs 735 | Lai 2020 |
| Anxiety | High risk vs low risk exposure | 1 year after outbreak:  Statistically significant:  4.9±4.3 vs 2.1±2.5  MD: 2.80, 95%CI [1.70;3.90]  (p<0.001)* | 71 vs 113 | McAlonan 2007 |
| Anxiety | Working in a high risk unit (vs working in a low risk unit) | Not statistically significant:  0.67±0.75 vs 0.57±0.46  MD: 0.10, 95%CI [-0.13;0.33]*  (p>0.05) | 65 vs 42 | Chen 2005 |
| Anxiety | High risk contact (vs no contact) | Statistically significant:  aOR: 2.062, 95%CI [1.349;3.153]  (p=0.001) | 2299 | Lu 2020 |
| Fear | High risk contact (vs no contact) | Statistically significant:  aOR: 1.408, 95%CI [1.025;1.933]  (p=0.034) | 2299 | Lu 2020 |
| **Depression-related symptoms** | | | | |
| Depression symptoms | Frontline vs second-line | Statistically significant:  94/522 vs 92/735  aOR: 1.52, 95%CI [1.11;2.09]  (p=0.01) | 522 vs 735 | Lai 2020 |
| Depression | Working in a high risk location | Not statistically significant:  aOR: 1.93, 95%CI [0.83; 4.50]  (p>0.05) | 549 | Liu 2012 |
| Depression | High risk contact (vs no contact) | Statistically significant:  aOR: 2.016, 95%CI [1.102;3.685]  (p=0.023) | 2299 | Lu 2020 |
| Depression | High risk vs low risk exposure | 1 year after outbreak:  Statistically significant:  4.9±4.8 vs 2.2±2.6  MD: 2.70, 95%CI [1.48;3.92]  (p<0.001)* | 71 vs 113 | McAlonan 2007 |
| Depression | Working in a high risk unit (vs working in a low risk unit) | Not statistically significant:  0.64±0.7 vs 0.55±0.47  MD: 0.09, 95%CI [-0.13;0.31]*  (p>0.05) | 65 vs 42 | Chen 2005 |
| **(Perceived) stress** | | | | |
| Perceived stress | High risk vs low risk exposure | During outbreak:  Not statistically significant:  17.0±5.7 vs 15.9±4.7  MD: 1.10, 95%CI [-0.44;2.64]  (p=0.18) * | 106 vs 70 | McAlonan 2007 |
| Perceived stress | High risk vs low risk exposure | Statistically significant:  β: 0.31, 95%CI [0.17;0.45]*  (p<0.0001) | 184 | McAlonan 2007 |
| Stress | High risk vs low risk exposure | 1 year after outbreak:  Statistically significant:  7.2±4.8 vs 4.1±3.2  MD: 3.10, 95%CI [1.84;4.36]  (p<0.001) * | 71 vs 113 | McAlonan 2007 |
| More stressed at work | SARS-affected hospitals vs non-SARS affected hospitals | Statistically significant:  aOR: 1.12, 95%CI [1.02;1.25] (inverse calculated)  (p<0.05) | 7614 | Koh 2005 |
| More stressed at work | Daily vs few times a week/rarely exposed to SARS | Statistically significant:  aOR: 1.33, 95%CI [1.19; 1.49] (inverse calculated)  (p<0.05) | 7614 | Koh 2005 |
| **Emotional exhaustion and burnout** | | | | |
| Emotional exhaustion | Contact with SARS patients | Statistically significant:  β: -0.15, 95%CI [-0.25;-0.05]*  (p=0.003)  (Low scores indicate greater contact) | 333 | Marjanovic 2007 |
| Burnout | Touched SARS patient | Not statistically significant (not entered in stepwise regression) | 587 | Maunder 2006 |
| Burnout | Worked in SARS unit | Not statistically significant (not entered in stepwise regression) | 587 | Maunder 2006 |
| Burnout | Ever in SARS patient room | Not statistically significant (not entered in stepwise regression) | 587 | Maunder 2006 |
| **Sleep problems** | | | | |
| Insomnia symptoms | Contact with COVID-19 patient | Not statistically significant:  aOR: 1.252, 95%CI [0.960;1.632]  (p=0.098) | 1563 | Zhang 2020 |
| Insomnia symptoms | Frontline vs second-line | Statistically significant:  64/522 vs 33/735  aOR: 2.97, 95%CI [1.92;4.60]  (p<0.001) | 522 vs 735 | Lai 2020 |
| **General symptoms of psychopathology** | | | | |
| Psychiatric morbidity | Contact with suspected SARS patient | Not statistically significant:  aOR: 0.51, 95%CI [0.17;1.50]  (p=0.22) | 277 | Sim 2004 |
| Psychiatric morbidity | Direct contact with SARS patients | Not statistically significant in logistic regression analysis | 652 | Tam 2004 |
| Psychiatric symptoms | Direct contact with SARS patients vs no direct contact with SARS patients | Not statistically significant:  aOR: 1.22, 95% CI [0.75;1.98]  (p=0.42) | 661 | Chan 2004 |
| Psychological distress | Touched SARS patient | Not statistically significant (not entered in stepwise regression) | 587 | Maunder 2006 |
| Psychiatric morbidity | Care for SARS patients | Not statistically significant (not entered in multiple regression) | 1203 (exact number not clear) | Chong 2004 |
| Psychological distress | Worked in SARS unit | Not statistically significant (not entered in stepwise regression) | 587 | Maunder 2006 |
| Psychological distress | Ever in SARS patient room | Not statistically significant (not entered in stepwise regression) | 587 | Maunder 2006 |
| Mental health | Total number of contact-hours with SARS patients | Statistically significant:  (p=0.038) | 90 | Chen 2007 |
| Psychiatric morbidity | Exposed to SARS | Statistically significant:  aOR: 1.62, 95%CI [1.1;2.4]  (p=0.017) | 1203 (exact number not clear) | Chong 2004 |
| Psychiatric morbidity | Work at fever room/tent | Not statistically significant:  aOR: 0.82, 85%CI [0.39;1.72]  (p=0.60) | 277 | Sim 2004 |
| Psychiatric morbidity | Frontline staff feedback reaching administrators | Not statistically significant in logistic regression analysis | 652 | Tam 2004 |
| Mental health | Exposure to COVID-19 (contact with patients or self/friend/family diagnosed) | Statistically significant:  β: 5.347, 95%CI [3.831;8.184]  (p<0.001) | 994 | Kang 2020 |
| **Anger** | | | | |
| State anger | Contact with SARS patients | Not statistically significant:  β: -0.09, 95%CI [-0.19;0.01]*  (p=0.068)  (Low scores indicate greater contact) | 333 | Marjanovic 2007 |
| **Being quarantined** | | | | |
| **Acute stress disorder/post-traumatic stress (symptoms)** | | | | |
| Acute stress disorder | Quarantine | Statistically significant:  aOR: 4.077, 95%CI [1.148;14.48]  (p<0.04) | 17 | Bai 2004 |
| Post-traumatic stress | Being quarantined | Statistically significant:  aOR: 2.09, 95%CI [1.00;4.37]  (p<0.05) | 549 | Wu 2009 |
| Post-traumatic stress | Being quarantined | Not statistically significant (not entered in stepwise regression) | 587 | Maunder 2006 |
| **Depression-related symptoms** | | | | |
| Depression | Any quarantining | Statistically significant:  aOR: 5.06, 95%CI [2.12, 12.10]  (p<0.05) | 549 | Liu 2012 |
| **Emotional exhaustion and burnout** | | | | |
| Emotional exhaustion | Time spent in quarantine | Not statistically significant:  β: 0.08, 95%CI [-0.02;0.18]*  (p=0.11) | 333 | Marjanovic 2007 |
| Burnout | Being quarantined | Not statistically significant (not entered in stepwise regression) | 587 | Maunder 2006 |
| **General symptoms of psychopathology** | | | | |
| Psychological distress | Being quarantined | Not statistically significant (not entered in stepwise regression) | 587 | Maunder 2006 |
| **Anger** | | | | |
| State anger | Time spent in quarantine | Statistically significant:  β: 0.14, 95%CI [0.04;0.24]*  (p=0.008) | 333 | Marjanovic 2007 |
| **Job stress and dissatisfaction** | | | | |
| **Acute stress disorder/post-traumatic stress (symptoms)** | | | | |
| Stress reactions | Being conscripted from a lower risk to a higher risk unit (vs working in low risk unit) | Statistically significant:  21.9±10.5 vs 13.7±9.1  MD: 8.2, 95%CI [2.9;13.5]*  (p<0.01) | 21 vs 42 | Chen 2005 |
| Psychological stress | Job stress | Statistically significant:  β: 0.208, 95%CI [0.162;0.254]*  (p<0.001) | 1557 | Maunder 2004 |
| Psychological stress | Dissatisfaction with hospital system and procedures | Not statistically significant:  β: -0.012, 95%CI [-0.058;0.034]*  (p=0.61) | 1557 | Maunder 2004 |
| Post-traumatic stress | Impact on work life | Statistically significant:  aOR: 1.9, 95%CI [1.3;2.7]  (p=0.001) | 248 | Styra 2008 |
| **Anxiety-related symptoms** | | | | |
| Anxiety | Being conscripted from a lower risk to a higher risk unit (vs working in low risk unit) | Not statistically significant:  0.75±0.51 vs 0.57±0.46  MD: 0.18, 95%CI [-0.08;0.44]*  (p>0.05) | 21 vs 42 | Chen 2005 |
| **Depression-related symptoms** | | | | |
| Depression | Job stress | Not statistically significant:  aOR: 0.60, 95%CI [0.16;2.19]  (p>0.05) | 549 | Liu 2012 |
| Depression | Being conscripted from a lower risk to a higher risk unit (vs working in low risk unit) | Statistically significant:  0.92±0.52 vs 0.55±0.47  MD: 0.37, 95%CI [0.11;0.63]*  (p<0.05) | 21 vs 42 | Chen 2005 |
| **(Perceived) stress** | | | | |
| Total stress load | Working hours per week | Statistically significant:  β: 0.841, 95% CI[0.041;1.641]*  (p=0.048) | 180 | Mo 2020 |
| **Emotional exhaustion and burnout** | | | | |
| MERS-related burnout | MERS-related job stress | Statistically significant:  β: 0.34, 95%CI [0.280;0.402]  (p<0.01) | 215 | Kim 2016 |
| **General symptoms of psychopathology** | | | | |
| Mental distress | Changes in work (transferred to another ward) | Not statistically significant in multiple linear regression model | 466 | Wong 2005 |
| Emotional distress | Precautionary measures affect ability to do job | Statistically significant:  aOR: 2.9, 95% [1.9;4.6]  (p<0.05) | 503 | Nickell 2004 |
| Psychiatric morbidity | Job-related stress | Statistically significant:  aOR: 4.06, 95%CI [2.28;7.23]  (p<0.05) | 652 | Tam 2004 |
| **Risk perception and health fear** | | | | |
| **Acute stress disorder/post-traumatic stress (symptoms)** | | | | |
| Post-traumatic symptoms | Fear of infection | Statistically significant:  β: 0.20, 95%CI [0.01;0.40]*  (p<0.05) | 97 | Ho 2005 |
| Psychological stress | Health fear | Statistically significant:  β: 0.205, 95%CI [0.148;0.262]*  (p<0.001) | 1557 | Maunder 2004 |
| Likelihood of post-traumatic stress disease | Perceived risk | Statistically significant:  HCW group:  β: 0.42  (p<0.01)  non-HCW group:  β: 0.17  (p<0.01) | 280 | Son 2019 |
| Post-traumatic stress | Perceived risk level | Statistically significant:  aOR: 2.40, 95%CI [2.05;2.81]  (p<0.05) | 549 | Wu 2009 |
| Post-traumatic stress | Perception of risk to others | Not statistically significant in multivariate logistical regression | 248 | Styra 2008 |
| Post-traumatic stress | Perception of personal risk | Statistically significant:  aOR: 2.0, 95%CI [1.4;2.8]  (p<0.001) | 248 | Styra 2008 |
| Symptomatic post-traumatic stress symptoms | Perceived negative feeling towards SARS | Statistically significant:  aOR: 11.1, 95%CI [2.3;52.9]  (p<0.005) | 70 | Su 2007 |
| **Anxiety-related symptoms** | | | | |
| Concern for personal or family health | Perceived death rate of SARS | Statistically significant:  5-9%:  aOR: 1.6, 95%CI [1.8;2.1]  (p<0.05)  ≥10%:  aOR: 5.0, 95%CI [2.6;9.6]  (p<0.05)  Don’t know:  aOR: 2.2, 95%CI [1.5;3.3]  (p<0.05) | 1958 | Nickell 2004 |
| **Depression-related symptoms** | | | | |
| Depression | Perception of SARS-related risks | Statistically significant:  aOR: 1.54, 95%CI [1.27, 1.86]  (p<0.0001) | 549 | Liu 2012 |
| **Emotional exhaustion and burnout** | | | | |
| MERS-related burnout | Fear of MERS-infection | Non statistically significant:  β: -0.01, 95%CI [-0.023;0.168]  (p=0.81) | 215 | Kim 2016 |
| **Sleep problems** | | | | |
| Insomnia | Perceived negative feeling towards SARS | Statistically significant:  aOR: 3.5, 95%CI [1.0;12.5]  (p<0.1) | 70 | Su 2007 |
| Insomnia symptoms | Worried about being infected | Statistically significant:  aOR: 2.299, 95%CI [1.573;3.360]  (p<0.001) | 1563 | Zhang 2019 |
| **General symptoms of psychopathology** | | | | |
| Mental distress | Health of self | Statistically significant:  β: 0.19  (p<0.05) | 466 | Wong 2005 |
| Mental distress | Spread of virus | Statistically significant:  β: 0.16  (p<0.05) | 466 | Wong 2005 |
| Mental distress | Health of family/others | Not statistically significant in multiple linear regression model | 466 | Wong 2005 |
| **Stigma** | | | | |
| **Acute stress disorder/post-traumatic stress (symptoms)** | | | | |
| Post-traumatic stress | Stigma and avoidance | Not statistically significant (not entered in stepwise regression analysis) | 587 | Maunder 2006 |
| Psychological stress | Social isolation and avoidance | Statistically significant:  β: 0.262, 95%CI [0.207;0.317]*  (p<0.001) | 1557 | Maunder 2004 |
| **Anxiety-related symptoms** | | | | |
| Concern for personal or family health | Being treated differently because of working in hospital | Statistically significant:  aOR: 1.6, 95%CI [1.2;2.1]  (p<0.05) | 1951 | Nickell 2004 |
| **(Perceived) stress** | | | | |
| Perceived stress | Stigma | Statistically significant:  β: 0.075, 95%CI [0.030;0.120]*  (p=0.002) | 187 | Park 2018 |
| **Emotional exhaustion and burnout** | | | | |
| Burnout | Stigma and avoidance | Not statistically significant (not entered in stepwise regression analysis) | 587 | Maunder 2006 |
| **General symptoms of psychopathology** | | | | |
| Mental health | Stigma | Statistically significant:  β: –0.306, 95%CI [-0.388;-0.224]*  (p<0.001)  (low scores on mental health scale mean worse mental health) | 187 | Park 2018 |
| Psychological distress | Stigma and avoidance | Not statistically significant (not entered in stepwise regression analysis) | 587 | Maunder 2006 |
| Mental distress | Being isolated (being discriminated/ alienated) | Not statistically significant in multiple linear regression model | 466 | Wong 2005 |
| **Loss of control and emotional disruption** | | | | |
| **Acute stress disorder/post-traumatic stress (symptoms)** | | | | |
| Post-traumatic symptoms | Insecurity | Statistically significant:  β: 0.52, 95%CI [0.34;0.70]*  (p<0.01) | 97 | Ho 2005 |
| Likelihood of post-traumatic stress disease | Negative emotional experience | Statistically significant:  HCW group:  β: 0.17  (p<0.05)  non-HCW group:  β: 0.30  (p<0.01) | 280 | Son 2019 |
| **General symptoms of psychopathology** | | | | |
| Mental distress | Vulnerability/loss of control | Statistically significant:  β: 0.43  (p<0.05) | 466 | Wong 2005 |
| **PROTECTIVE FACTORS** | | | | |
| **Organizational communication and support** | | | | |
| **Acute stress disorder/post-traumatic stress (symptoms)** | | | | |
| Post-traumatic stress | Clear communication of directives and precautionary measures | Not statistically significant:  aOR: 0.81, 95% CI [0.37;1.74]  (p=0.58) | 661 | Chan 2004 |
| Post-traumatic stress | Being able to give feedback to hospital management and support from hospital administration | Not statistically significant:  aOR: 1.1, 95% CI [0.54;2.14]  (p=0.83) | 661 | Chan 2004 |
| Post-traumatic stress | Confidence in the information provided | Not statistically significant in multivariate logistical regression | 248 | Styra 2008 |
| **Anxiety-related symptoms** | | | | |
| Anxiety level | SARS prevention program | Before vs 2 weeks after program initiation:  Statistically significant:  β: -0.2338, 95%CI [-0.4051;-0.0625]  (p=0.0075) | 116 | Chen 2006 |
| Anxiety level | SARS prevention program | Before vs 1 month after program initiation:  Statistically significant:  β: -0.2967, 95%CI [-0.4272; -0.1661  (p<0.0001) | 116 | Chen 2006 |
| Anxiety level | SARS prevention program | Before vs 3 months after caring:  Statistically significant:  β: -0.4135, 95%CI [-0.5366;-0.2904]  (p<0.0001) | 116 | Chen 2006 |
| **Depression-related symptoms** | | | | |
| Depression level | SARS prevention program | Before vs 2 weeks after program initiation:  Statistically significant:  β: -0.4598, 95%CI [-0.6567;-0.2628]  (p<0.0001) | 116 | Chen 2006 |
| Depression level | SARS prevention program | Before vs 1 month after program initiation:  Statistically significant:  β: -0.3662, 95%CI [-0.5158;-0.2166]  (p<0.0001) | 116 | Chen 2006 |
| Depression level | SARS prevention program | Before vs 3 months after caring:  Statistically significant:  β: -0.4784, 95%CI [-0.6260;-0.3315]  (p<0.0001) | 116 | Chen 2006 |
| **Emotional exhaustion and burnout** | | | | |
| Emotional exhaustion | Organizational support | Not statistically significant:  β: 0.04, 95%CI [-0.05;0.13]*  (p=0.41)  (Low scores indicate greater organizational support) | 333 | Marjanovic 2007 |
| **Sleep problems** | | | | |
| Sleep quality level | SARS prevention program | Before vs 2 weeks after program initiation:  Statistically significant:  β: -2.2959, 95%CI [-3.9108;-0.6810]  (p=0.0053) | 116 | Chen 2006 |
| Sleep quality level | SARS prevention program | Before vs 1 month after program initiation:  Statistically significant:  β: -1.9193, 95%CI [-3.1163;-0.7223]  (p=0.0017) | 116 | Chen 2006 |
| Sleep quality level | SARS prevention program | Before vs 3 months after caring:  Statistically significant:  β: -2.0217, 95%CI [-3.1986;-0.8448]  (p=0.0008) | 116 | Chen 2006 |
| **General symptoms of psychopathology** | | | | |
| Psychiatric morbidity | Adequate counseling and psychological support from employer | Statistically significant:  aOR: 0.53, 95%CI [0.31;0.89]  (p<0.05) | 652 | Tam 2004 |
| Psychiatric morbidity | Adequate insurance and compensation | Statistically significant:  aOR: 0.52, 95%CI [0.29;0.93]  (p<0.05) | 652 | Tam 2004 |
| Psychiatric morbidity | Expressing opinions through staff unions or mass media | Not statistically significant in logistic regression analysis | 652 | Tam 2004 |
| Psychiatric symptoms | Clear communication of directives and precautionary measures | Statistically significant:  aOR: 0.51, 95% CI [0.29;0.90]  (p=0.020) | 661 | Chan, 2004 |
| Psychiatric symptoms | Being able to give feedback to hospital management and support from hospital administration | Not statistically significant:  aOR: 0.86, 95% CI [0.44;1.69]  (p=0.67) | 661 | Chan 2004 |
| Mental health | Accessed mental health care services | Statistically significant:  β: -0.868, 95%CI [-1.385;-0.289]  (p=0.001) | 994 | Kang 2020 |
| **Anger** | | | | |
| State anger | Organizational support | Statistically significant:  β: 0.24, 95%CI [0.13;0.35]*  (p=0.000)  (Low scores indicate greater organizational support) | 333 | Marjanovic 2007 |
| **Physical safety and training** | | | | |
| **Acute stress disorder/post-traumatic stress (symptoms)** | | | | |
| Psychological stress | Doubt about protection | Not statistically significant:  β: -0.035, 95%CI [-0.082;0.012]*  (p=0.14) | 1557 | Maunder 2004 |
| Post-traumatic stress | Confidence in infection control measures | Not statistically significant in multivariate logistical regression | 248 | Styra 2008 |
| Post-traumatic stress | Perceived adequacy of training, protection and support | Statistically significant:  β: -0.22, 95%CI [-0.38;-0.06]*  (p=0.01) | 587 | Maunder 2006 |
| Post-traumatic stress | Unprotected exposure | Not statistically significant (not entered in stepwise regression) | 587 | Maunder 2006 |
| **Anxiety-related symptoms** | | | | |
| Concern for personal or family health | Precautionary measures sufficient | Statistically significant:  aOR: 0.4, 95%CI [0.3;0.5]  (p<0.05) | 1969 | Nickell 2004 |
| Anxiety | Formal training in handling infectious diseases in primary care | Statistically significant:  aOR: 5.41, 95%CI [1.02;28.79]  (p=0.048) | 50 | Wong 2007 |
| **Emotional exhaustion and burnout** | | | | |
| Emotional exhaustion | Trust in equipment/infection control initiative | Statistically significant:  β: -0.15, 95%CI [-0.26;-0.05]*  (p=0.005) | 333 | Marjanovic 2007 |
| Burnout | Perceived adequacy of training, protection and support | Statistically significant:  β: -0.27, 95%CI [-0.44;-0.10]*  (p=0.002) | 587 | Maunder 2006 |
| Burnout | Unprotected exposure | Not statistically significant (not entered in stepwise regression) | 587 | Maunder 2006 |
| **Sleep problems** | | | | |
| Insomnia symptoms | Received sufficient infection prevention training | Not statistically significant:  aOR: 1.029, 95%CI [0.782;1.354]  (p=0.838) | 1563 | Zhang 2020 |
| Insomnia symptoms | Current protection can prevent getting infected | Not statistically significant:  aOR: 0.787, 95%CI [0.597;1.037]  (p=0.089) | 1563 | Zhang 2020 |
| Insomnia symptoms | Uncertainty regarding effective disease control (very strong vs no feeling) | Statistically significant:  aOR: 3.297, 95%CI [1.284;8.469]  (p=0.013) | 1563 | Zhang 2020 |
| **General symptoms of psychopathology** | | | | |
| Psychiatric morbidity | Clear infection control guidelines | Not statistically significant in logistic regression analysis | 652 | Tam 2004 |
| Psychiatric morbidity | Protective facilities and temporary residential arrangements | Not statistically significant in logistic regression analysis | 652 | Tam 2004 |
| Psychological disorder | Perception of being adequately trained and supported by the hospital | Statistically significant:  β: -0.20  (p=0.03) | 133 | Lancee 2008 |
| Psychological distress | Perceived adequacy of training, protection and support | Not statistically significant (not entered in stepwise regression) | 587 | Maunder 2006 |
| Psychological distress | Unprotected exposure | Not statistically significant (not entered in stepwise regression) | 587 | Maunder 2006 |
| **Anger** | | | | |
| State anger | Trust in equipment/infection control initiative | Statistically significant:  β: -0.14, 95%CI [-0.25;-0.03]*  (p=0.011) | 333 | Marjanovic 2007 |
| **Social support** | | | | |
| **Acute stress disorder/post-traumatic stress (symptoms)** | | | | |
| Post-traumatic stress | Support from supervisors and colleagues | Statistically significant:  aOR: 0.33, 95% CI [0.16;0.69]  (p=0.003) | 661 | Chan 2004 |
| Post-traumatic stress | Support from family | Not statistically significant:  aOR: 0.82, 95% CI [0.35;1.94]  (p=0.65) | 661 | Chan 2004 |
| Post-traumatic stress | Being able to talk to someone about my concerns | Not statistically significant:  aOR: 1.13, 95% CI [0.55;2.30]  (p=0.75) | 661 | Chan 2004 |
| **Anxiety-related symptoms** | | | | |
| Anxiety | Social support | Statistically significant:  β: -0.781, 95%CI [-0.948;-0.614]*  (p<0.0001) | 180 | Xiao 2020 |
| **(Perceived) stress** | | | | |
| Stress | Social support | Statistically significant:  β: -0.704, 95%CI [-1.161;-0.247]*  (p=0.003) | 180 | Xiao 2020 |
| Self-efficacy | Social support | Statistically significant:  β: 0.023, 95%CI [0.011;0.035]*  (p<0.001) | 180 | Xiao 2020 |
| **Emotional exhaustion and burnout** | | | | |
| Emotional exhaustion | Social interaction | Not statistically significant:  β: -0.03, 95%CI [-0.17;0.11]*  (p>0.05) | 211 | Chang 2006 |
| Emotional exhaustion | Trust (to be able to rely on colleagues) | Statistically significant:  β: -0.26, 95%CI [-0.40;-0.12]*  (p<0.001) | 211 | Chang 2006 |
| MERS-related burnout | Support from family and friends | Statistically significant:  β: -0.14, 95%CI [-0.216;-0.064]  (p<0.01) | 215 | Kim 2016 |
| **Sleep problems** | | | | |
| Sleep quality | Social support | Not statistically significant:  β: –0.020, 95%CI [-0.111;0.019]*  (p=0.54) | 180 | Xiao 2020 |
| **General symptoms of psychopathology** | | | | |
| Psychiatric morbidity | Sense of coherence and team spirit | Not statistically significant in logistic regression analysis | 652 | Tam 2004 |
| Psychiatric morbidity | Appreciation from the community | Not statistically significant in logistic regression analysis | 652 | Tam 2004 |
| Psychiatric morbidity | Support from relatives | Not statistically significant in logistic regression analysis | 652 | Tam 2004 |
| Psychiatric symptoms | Support from supervisors and colleagues | Statistically significant:  aOR: 0.35, 95% CI [0.17;0.69]  (p=0.003) | 661 | Chan 2004 |
| Psychiatric symptoms | Support from family | Not statistically significant:  aOR: 0.83, 95% CI [0.38;1.84]  (p=0.65) | 661 | Chan 2004 |
| Psychiatric symptoms | Being able to talk to someone about my concerns | Not statistically significant:  aOR: 1.07, 95% CI [0.56;2.04]  (p=0.84) | 661 | Chan 2004 |
| **Sense of control and coping** | | | | |
| **Acute stress disorder/post-traumatic stress (symptoms)** | | | | |
| Post-traumatic symptoms | Perceived self-efficacy | Not statistically significant:  β: -0.02, 95%CI [-0.08;0.04]*  (p>0.05) | 97 | Ho 2005 |
| Likelihood of post-traumatic stress disease | Coping ability | HCW group:  Not statistically significant  β: -0.13  (p>0.05)  non-HCW group:  Statistically significant:  β: -0.18  (p< 0.01) | 280 | Son 2019 |
| Post-traumatic morbidity | Coping strategy: Self-distraction | Statistically significant:  aOR: 1.75, 95%CI [1.09;2.80]  (p=0.02) | 277 | Sim 2004 |
| Post-traumatic morbidity | Coping strategy: Instrumental support seeking | Not statistically significant:  aOR: 1.84, 95%CI [0.91;3.72]  (p=0.09) | 277 | Sim 2004 |
| Post-traumatic morbidity | Coping strategy: Behavioural disengagement | Statistically significant:  aOR: 2.85, 95%CI [1.25;6.47]  (p=0.01) | 277 | Sim 2004 |
| Post-traumatic morbidity | Coping strategy: Venting | Statistically significant:  aOR: 0.51, 95%CI [0.26;0.97]  (p=0.04) | 277 | Sim 2004 |
| Post-traumatic morbidity | Coping strategy: Humor | Statistically significant:  aOR: 0.45, 95%CI [0.21;0.97]  (p=0.04) | 277 | Sim 2004 |
| Post-traumatic morbidity | Coping strategy: Acceptance | Statistically significant:  aOR: 0.53, 95%CI [0.32;0.89]  (p=0.02) | 277 | Sim 2004 |
| Post-traumatic morbidity | Coping strategy: Religion | Statistically significant:  aOR: 0.70, 95%CI [1.20;2.39]  (p=0.003) | 277 | Sim 2004 |
| Post-traumatic stress | Religious convictions | Not statistically significant:  aOR: 1.52, 95% CI [0.88;2.62]  (p=0.14) | 661 | Chan 2004 |
| Post-traumatic morbidity | Coping strategy: Active coping | Not statistically significant:  aOR: 0.84, 95%CI [0.45;1.54]  (p=0.57) | 277 | Sim 2004 |
| Post-traumatic morbidity | Coping strategy: Denial | Not statistically significant:  aOR: 0.84, 95%CI [0.50;1.44]  (p=0.53) | 277 | Sim 2004 |
| Post-traumatic morbidity | Coping strategy: Emotional support seeking | Not statistically significant:  aOR: 0.92, 95%CI [0.52;1.62]  (p=0.76) | 277 | Sim 2004 |
| Post-traumatic morbidity | Coping strategy: Positive reframing | Not statistically significant:  aOR: 1.09, 95%CI [0.71;1.66]  (p=0.70) | 277 | Sim 2004 |
| Post-traumatic morbidity | Coping strategy: Planning | Not statistically significant:  aOR: 1.35, 95%CI [0.72;2.53]  (p=0.36) | 277 | Sim 2004 |
| Post-traumatic stress | Altruistic acceptance | Statistically significant:  aOR: 0.47, 95%CI [0.25;0.89]  (p<0.05) | 549 | Wu 2009 |
| Post-traumatic stress | Maladaptive coping | Statistically significant:  β: 0.37, 95%CI [0.20;0.54]*  (p<0.001) | 587 | Maunder 2006 |
| Post-traumatic stress | Adaptive coping | Not statistically significant (not entered in stepwise regression analysis) | 587 | Maunder 2006 |
| **Anxiety-related symptoms** | | | | |
| Anxiety | Positive coping | Statistically significant:  r: -0.182  (p=0.002) | 165 | Zhu 2020 |
| **Depression-related symptoms** | | | | |
| Depression | Altruistic acceptance | Statistically significant:  aOR: 0.26, 95%CI [0.12;0.56]  (p<0.05) | 549 | Liu 2012 |
| Depression | Positive attitude towards SARS | Statistically significant:  aOR: 12.7, 95%CI [1.1;150]  (p<0.05) | 70 | Su 2007 |
| Depression | Positive coping | Statistically significant:  r: -0.253  (p=0.001) | 165 | Zhu 2020 |
| **(Perceived) stress** | | | | |
| Perceived stress | Hardiness | Statistically significant:  β: –0.310, 95%CI [-0.410;0.210]*  (p<0.001) | 187 | Park 2018 |
| **Emotional exhaustion and burnout** | | | | |
| Emotional exhaustion | Levels of vigor | Statistically significant:  β: -0.34, 95%CI [-0.44;-0.24]*  (p=0.000) | 333 | Marjanovic 2007 |
| Burnout | Maladaptive coping | Statistically significant:  β: 0.29, 95%CI [0.12;0.46]*  (p=0.001) | 587 | Maunder 2006 |
| Burnout | Adaptive coping | Not statistically significant (not entered in stepwise regression analysis) | 587 | Maunder 2006 |
| **General symptoms of psychopathology** | | | | |
| Mental health | Hardiness | Statistically significant:  β: 0.487, 95%CI [0.291;0.683]*  (p<0.001)  (low scores on mental health scale mean worse mental health) | 187 | Park 2018 |
| Psychological distress | Maladaptive coping | Statistically significant:  β: 0.31, 95%CI [0.15;0.47]*  (p<0.001) | 587 | Maunder 2006 |
| Psychological distress | Adaptive coping | Not statistically significant (not entered in stepwise regression analysis) | 587 | Maunder 2006 |
| Psychiatric symptoms | Religious convictions | Not statistically significant:  aOR: 0.86, 95% CI [0.55;1.36]  (p=0.53) | 661 | Chan 2004 |
| **Anger** | | | | |
| State anger | Levels of vigor | Statistically significant:  β: -0.20, 95%CI [-0.31;-0.09]*  (p=0.000) | 333 | Marjanovic 2007 |

aOR: adjusted odds ratio, CI: confidence interval, MD: mean difference, β: regression coefficient, r: correlation coefficient

* Calculations done by the reviewers
