# Supplementary material for: Factors affecting mental health of health care workers during coronavirus disease outbreaks (SARS, MERS & COVID-19): A rapid systematic review
Source: PLoS One. 2020 Dec 15;15(12):e0244052. doi: 10.1371/journal.pone.0244052 (PMC7737991; doi:10.1371/journal.pone.0244052)
Supplement: S4 Table — (DOCX) [file pone.0244052.s004.docx]

**S5 Table. Risk of bias assessment**

| **Author, Year** | **Inappropriate eligibility criteria** | **Inappropriate methods for exposure and outcome variables** | **Not controlled for confounding** | **Incomplete or inadequate follow-up** | **Other limitations** |
| --- | --- | --- | --- | --- | --- |
| Bai, 2004 | No, survey was sent after all members from a single hospital returned to work and the response rate was 61%. | Yes, cross-sectional study design with questionnaires | No, stepwise multiple logistic regression model to adjust for related factors for acute stress disorder | N/A | No, only some concerns regarding recall bias since survey was completed within one month after the hospitalization of SARS patients  Yes, serious concerns regarding reporting bias since info on model development and inclusion of variables is lacking |
| Chan, 2004 | No, survey was sent to all doctors and nurses from a single hospital by email and the response rate was 67%. | Yes, cross-sectional study design with questionnaires | No, logistic regression analysis was performed to adjust for the predictors of psychiatric symptoms. | N/A | No, only some concerns regarding recall bias since survey was completed 2 months after the first case of SARS was reported in Singapore |
| Chang, 2006 | No, survey was sent to medical professionals in four medical centres and the response rate was 61% (53% successfully completed). | Yes, cross-sectional study design with questionnaires | No, multiple regression analyses to predict the impact of social interaction and trust on emotional exhaustion | N/A | No, only some concerns regarding recall bias since survey was completed 2-3 months after the first case of  SARS was reported in Taiwan  Other reported limitations: Cultural differences also influence the  creation of social capital. Factors such as deference to authority, stoicism, fatalism, societal cohesiveness, and social homogeneity could affect how trust and social capital are built and maintained. |
| Chen, 2005 | No, survey was sent to nurses working with patients in wards or emergency units in a single hospital hospital (no random sampling) and the response rate was 70%. | Yes, cross-sectional study design with questionnaires | No, controlled for demographic variables by analyses of covariance (ANCOVAs) followed by post hoc least-significant-difference comparisons to compare symptoms across three groups of nurses | N/A | No, only some concerns regarding recall bias since survey was completed at the peak of the SARS outbreak |
| Chen, 2006 | Unclear, survey was sent to nursing staff from largest obligatory SARS designated treatment hospital in Taiwan but there was no further info on the selection procedure. Info on the response rate is lacking. | Yes, outcome measures were assessed with questionnaires | No, GEE analysis was performed to control for changes in time and possible confounding factors such as perceived  work stress before the SARS epidemic and level of family support while performing SARS patient care. | No, drop-out rate of 3.3% | No, only some concerns regarding recall bias since survey was completed when nurses were caring for SARS patients at several time points |
| Chen, 2007 | Unclear, survey was sent to HCWs who treated SARS patient and control group (hospital staff with no SARS contacts) from a single hospital but there was no further info on the selection procedure. Info on the response rate is lacking. | Yes, cross-sectional study design with questionnaires | No, multivariable regression models were applied to adjust for the risk factors. | N/A | No, only some concerns regarding recall bias since survey was completed during the period when SARS was endemic |
| Chong, 2004 | No, survey was sent to all staff from a single hospital who were actually on service during  the outbreak and response rate was 52% (50% successfully completed) | Yes, cross-sectional study design with questionnaires | No, multivariate regression model was performed to adjust for significant variables in the univariate analysis. | N/A | No, only some concerns regarding recall bias since survey was completed at the time when the hospital was officially declared as having a serious nosocomial infection with SARS  Other reported limitations: only about half of the HCWs who were exposed to the threat responded in the study. |
| Ho, 2005 | Yes, the survey was sent to staff members who had been infected by SARS but are now recovering (regression analysis). The participants were not only HCWs but also patients. The response rate was only 30%. | Yes, cross-sectional study with questionnaires | No, multiple regression analysis was performed to adjust for perceived self-efficacy, infection, insecurity, and instability. | N/A | Yes, serious concerns on recall bias since survey was completed 3-4 months after infection  Other reported limitations: Sample included fewer doctors but more allied health professionals and support staff than respective proportions among non-respondents. |
| Kang, 2020 | Yes, internet-mediated questionnaire recruiting doctors and nurses in all workstations in Wuhan involves self-selection and it is uncertain whether the sample is representative for the study population. | Yes, cross-sectional study with questionnaires | No, a structural equation model was constructed to explore the relationship among the four main components, namely,  exposure, accessed mental healthcare services, mental health status and self-perceived health status  compared to that before the COVID-19 outbreak. | N/A | No, only some concerns regarding recall bias since survey was completed during the COVID-19 outbreak in Wuhan. |
| Kim, 2016 | No, survey was sent to nurses recruited from eight institutions by simple random sampling. The response rate was 92.9% (90% successfully completed). | Yes, cross-sectional study with questionnaires | No, multiple regression was performed to adjust for significant variables in the difference testing and correlation analysis. | N/A | No, only some concerns regarding recall bias since survey was completed when the disease had not yet been controlled |
| Koh, 2005 | No, survey was sent to all employees  on the payroll of nine healthcare  institutions and the response rate was 70%. | Yes, cross-sectional study with questionnaires | No, multiple logistic  regression was performed to adjust for each of the independent variables. | N/A | Yes, serious concerns regarding recall bias since the survey was completed towards the tail end of the epidemic |
| Lai, 2020 | No, 34 hospitals were selected based on region-stratified, 2-stage cluster sampling and one clinical department was randomly sampled from each selected hospital. The survey was sent to all HCWs in this department and the response rate was 68.7%. | Yes, cross-sectional study with questionnaires | No, multivariable logistic regression analysis was performed to adjust for confounders including sex, marital status, educational level, technical title, place of residence, working position and type of hospital. | N/A | No, only some concerns regarding recall bias since the survey was completed during the period that the total confirmed  cases of COVID-19 exceeded 10000 in China |
| Lancee, 2008 | Unclear, survey was sent to HCWs in nine hospitals but there was no further info on the selection procedure. The info on response rate is lacking (23 to 24% of total sample also participated in a diagnostic interview). | Unclear, cross-sectional studies with questionnaires but diagnostic interviews were also performed | No, logistic regression analysis was performed to adjust for previous episodes of a psychiatric  disorder, perception of adequate training and support, and years of health care experience. | N/A | Yes, serious concerns regarding recall bias since the survey was completed in the one- to two-year  period after the outbreak |
| Liu, 2019 | No, survey was sent to a random sample (stratified by profession and age group) of the employees of SARS affected hospital and the response rate was 83%. | Yes, cross-sectional study with questionnaires | No, multinomial logistic regression analysis was performed to adjust for socio-demographic factors, PTSD symptom level and job variables. | N/A | Yes, serious concerns regarding recall bias since the survey was completed three years after Beijing’s SARS outbreak  Other reported limitations:  Doctors and nurses falling into the high work exposure  category were oversampled. Hospital employees aged 35 to 55 were also oversampled. |
| Lu, 2020 | No, a questionnaire was sent to medical workforce of a single hospital fighting against COVID-19. The staff participated in epidemic prevention and control work for a month. The response rate was 94.88% | Yes, cross-sectional study with questionnaires | No, multivariate analyses for anxiety and depression were performed to adjust for gender, working years, native place, fertility status, and days of working in the isolation ward. | N/A | No, only some concerns regarding recall bias since survey was completed during the COVID-19 outbreak in China. |
| Marjanovic, 2007 | Yes, internet-mediated questionnaire recruiting registered nurses involves self-selection and it is uncertain whether the internet sample is representative for the study population. | Yes, cross-sectional study with questionnaires | No, three linear multiple regression analysis was performed to adjust for risk factors. | N/A | Yes, serious concerns regarding recall bias since the survey was completed about one year after the SARS outbreak  Other reported limitations:  The models accounted only for 25-26% of the variance. |
| Maunder, 2004 | Yes, survey was sent to all personnel of three hospitals and the response rate was only 10.5%.  Despite the low response rate, the  characteristics of the study sample are similar to the total population of the institutions surveyed. | Yes, cross-sectional study with questionnaires | No, regression analyses was performed to adjust for all variables simultaneously. | N/A | No, only some concerns regarding recall bias since the survey was completed during (at the end of) the outbreak  Other reported limitations:  There were differences in sampling methods between the three hospitals (lobby display/intranet vs email) |
| Maunder, 2006 | Yes, survey was sent to HCWs, including nurses in medical  and surgical inpatient units and all staff of intensive care units, emergency departments, and SARS isolation units, from 13 hospitals but there was no further info on the selection procedure. The response rate was only 39%. | Yes, cross-sectional study with questionnaires | No, stepwise regression model was performed to adjust for significant variables in the univariate analyses. | N/A | Yes, serious concerns regarding recall bias since the survey was completed in the one- to two-year period after the outbreak  Yes, serious concerns regarding reporting bias since only the significant variables were reported.  Other reported limitation:  HCWs who  had contact with SARS patients are oversampled in the  study, which may be because the study had greater salience for those persons. |
| McAlonen, 2007 | Unclear, survey was sent to HCWs, including doctors,  nurses, and health care assistants, from 2 acute care hospitals but there was no further info on the selection procedure. Info on the response rate is lacking. | Yes, cross-sectional study with questionnaires | No, groups in univariate analyses were matched on age, sex and educational level and hierarchical multiple regression analyses were performed based on Baron and Kenny’s Criteria to test the mediation model. | N/A | No, only some concerns regarding recall bias since the survey was completed at the peak of the outbreak. However, the psychological state of the HCW was also assessed by questionnaires one year after the outbreak. |
| Mo, 2020 | No, nurses from Guanxi supporting Wuhan in fighting against COVID-19 were selected by convenience sampling. The response rate was 85.71%. | Yes, cross-sectional study with questionnaires | No, multivariate analysis was performed to adjust for significant variables in the univariate analyses. | N/A | No, only concerns regarding recall bias since the survey was completed towards during the COVID-19 outbreak in Wuhan. |
| Nickell, 2004 | Yes, survey was distributed to all willing employees entering the hospital between 5:45 am and 7:15 pm (covering all major shift changes) over a 3-day period but the response rate was only 47%. | Yes, cross-sectional study with questionnaires | No, stepwise logistic regression analysis  was performed to  adjust for other identified explanatory variables. | N/A | No, only some concerns regarding recall bias since the survey was completed during the outbreak  Yes, there were serious concerns regarding reporting bias. Variables were determined to contribute to the model if the significance level for the Wald inclusion test statistic was less than 0.05 but results of the univariate analyses are lacking.  Other reported limitations:  Staff who were in quarantine or away because of illness or vacation were not included; some of these people may have been among those most concerned and most affected by the outbreak. |
| Park, 2017 | Unclear, the survey was sent to nurses working in high-risk areas in one hospital. They were selected based a list of potential participants provided by the  nursing administration office. Info on the response rate is lacking. | Yes, cross-sectional study with questionnaires | No, the direct and indirect effects were computed using a series of ordinary least-squares regressions and 95% bootstrap confidence intervals (95% Boot CIs) with 10,000 bootstrap resamples from the data. | N/A | No, only some concerns regarding recall bias since the survey was completed during the outbreak  Other reported limitations:  The effect size was calculated as the ratio of an indirect effect to a direct effect. This approach is currently the most widely used measure; however, a very large sample is required to obtain stable estimations of this ratio measure. |
| Sim, 2004 | No, survey was sent to doctors and nurses within a network of 9 polyclinics via the clinic operation manager. All medical staff received an earlier notification email. The response rate was 92%. | Yes, cross-sectional study with questionnaires | No, multiple logistic regression analyses were performed to adjust for socio-demographic variables, work-related items, contact with SARS and coping strategies. | N/A | Yes, serious concerns on recall bias since survey was completed some months after Singapore was removed from list of areas with recent transmissions by WHO  Other reported limitations: Other factors that would contribute to the psychological response to traumatic stress, such as personality variables, past trauma, or precise details of social support, were not  examined in this study. |
| Son, 2019 | Unclear, survey was sent to HCW and non-HCW from a single hospital but there was no further info on the selection procedure. Info on the response rate was lacking. | Yes, cross-sectional study with questionnaires | No, path analysis was performed to examine direct and indirect effects of precedent factors. | N/A | Yes, serious concerns on recall bias since survey was completed about 1 month after the end of the outbreak announced by the public health authority  Factors such as  the availability of resources from a hospital or community may enhance hospital workers’ resilience and were not examined in this study. |
| Styra, 2008 | Yes, after approval by each unit manager from a single hospital, the survey was sent to HCW working in their units but there was no further info on the selection procedure. The response rate was only 41.1%. | Yes, cross-sectional study with questionnaires | No, multivariate logistic regression analysis was performed to adjust for different risk factors. | N/A | Yes, serious concerns on recall bias since survey was completed about 1-2 months after Toronto was removed from the list of areas with recent transmission by WHO  Other reported limitations:  Survey responses from the high-risk units were higher than those from the low-risk comparison units. |
| Su, 2007 | Unclear, survey was sent to nurses from a single hospital but there was no further info on the selection procedure. Info on the response rate was lacking. 95.3% of the recruited nurses successfully completed the study. | Unclear, cross-sectional study with questionnaires but also with face-to-face interview-based diagnosis (MINI) | No, multiple logistic  regression analyses were performed to adjust for the different risk factors. | N/A | No, only some concerns regarding recall bias since survey was completed during the acute phase of the outbreak  However, objective MINI diagnosis  were obtained retrospectively, within a month after the  study was finished. The results did not reflect the real  time psychiatric morbidity. |
| Tam, 2004 | Yes, ward managers and department heads were contacted to obtain lists of their staff members and survey was sent to these HCW from three hospitals and response rate was only 40.2%. | Yes, cross-sectional study with questionnaires | No, forward conditional logistic regression analysis was performed to adjust for the different risk factors. | N/A | Yes, serious concerns regarding recall bias since the survey was completed near the end of the epidemic |
| Wong, 2005 | Yes, survey was sent to all doctors, nurses and healthcare assistants working in the emergency departments of 14 public hospitals. A contact person in each department was responsible for the distribution and collection of the questionnaires and the response rate was only 37%. | Yes, cross-sectional study with questionnaires | No, multiple linear regression analysis was performed to adjust for different sources of distress. | N/A | Yes, serious concerns regarding recall bias since the survey was completed at a time when Hong Kong was no longer an affected area, and the full impact of SARS at its peak could not be captured |
| Wong, 2007 | No, survey was sent to family medicine tutors or doctors from two universities but there was no further info on the selection procedure. The response rate was 74.8% in Hong Kong. Of note, in Toronto, only 34.0% of the doctors replied. | Yes, cross-sectional study with questionnaires | No, multiple logistic regression analysis was performed to adjust for risk factors. | N/A | No, only some concerns regarding recall bias since survey was completed during the outbreak |
| Wu, 2009 | No, survey was sent to randomly selected employees from a single hospital and the response rate was 83%. | Yes, cross-sectional study with questionnaires | No, logistic regression analyses were subsequently conducted in 3 steps while adding different risk factors to the model. | N/A | Yes, serious concerns regarding recall bias since the survey was completed three years after Beijing’s SARS outbreak |
| Xiao, 2020 | No, survey was sent to doctors or nurses who worked in departments of respiratory medicine, fever clinics, or the intensive care unit but there was no further info on the selection procedure. The response rate was: 81.82%. | Yes, cross-sectional study with questionnaires | No, multivariate analysis using the structural equation model with path analysis were performed to determine the structural relationship between the measured risk factors. | N/A | No, only some concerns regarding recall bias since the survey was completed during COVID-19 outbreak |
| Zhang, 2020 | Yes, internet-mediated questionnaire recruiting hospital staff using WeChat from all over China involves self-selection and it is uncertain whether the sample is representative for the study population. | Yes, cross-sectional study with questionnaires | No, multiple binary logistic regression analysis was performed to examine the associations between demographic factors and insomnia. Significant  differences were all included in the regression model except for  parameters with high collinearity. | N/A | No, only some concerns regarding recall bias since the survey was completed during COVID-19 outbreak. |
| Zhu, 2020 | Yes, internet-mediated questionnaire recruiting first line medical staff from Gansu province involves self-selection and it is uncertain whether the sample is representative for the study population. Information was collected through friend circle forwarding and WeChat group promotion. | Yes, cross-sectional study with questionnaires | No, partial correlation analysis was performed to control for gender and history of depression or anxiety. | N/A | No, only some concerns regarding recall bias since the survey was completed during COVID-19 outbreak. |
