# Supplementary material for: Factors affecting mental health of health care workers during coronavirus disease outbreaks (SARS, MERS & COVID-19): A rapid systematic review
Source: PLoS One. 2020 Dec 15;15(12):e0244052. doi: 10.1371/journal.pone.0244052 (PMC7737991; doi:10.1371/journal.pone.0244052)
Supplement: S1 Text — (DOCX) [file pone.0244052.s005.docx]

**MEDLINE (via the PubMed interface)**

“healthcare professional”[TIAB] OR “healthcare professionals”[TIAB] OR "Caregivers"[Mesh] OR caregiver*[TIAB] OR “care provider”[TIAB] OR “care providers”[TIAB] OR "Physicians"[Mesh] OR physician*[TIAB] OR doctor*[TIAB] OR "Nurses"[Mesh] OR nurse*[TIAB] OR "Personnel, Hospital"[Mesh] OR "Allied Health Personnel"[Mesh] OR "Medical Laboratory Personnel"[Mesh] OR "Medical Staff"[Mesh] OR “medical staff”[TIAB] OR “medical workers”[TIAB] OR “health workers”[TIAB] OR “health professionals”[TIAB] OR “healthcare workers”[TIAB] OR “medical personnel”[TIAB] OR paramedic*[TIAB] OR volunteer*[TIAB] OR “General Practitioners”[Mesh] OR “general practitioner”[TIAB] OR “general practitioners”[TIAB] OR “first responder”[TIAB] OR “emergency medical technicians”[TIAB]

AND

"Epidemics"[Mesh] OR epidemic*[TIAB] OR "Disease Outbreaks"[Mesh:NoExp] OR outbreak[TIAB] OR pandemic*[TIAB] OR “public health emergency”[TIAB] OR "SARS Virus"[Mesh] OR SARS[TIAB] OR “Middle East Respiratory Syndrome Coronavirus”[Mesh] OR MERS[TIAB] OR "Ebolavirus"[Mesh] OR ebola[TIAB] OR "Zika Virus"[Mesh] OR "Zika Virus Infection"[Mesh] OR zika[TIAB] OR "Coronavirus Infections"[Mesh] OR “corona virus”[TIAB] OR covid-19[TIAB]

AND

"Mental Health Services"[Mesh:NoExp] OR "Counseling"[Mesh] OR counseling[TIAB] OR “psychological assistance”[TIAB] OR “psychological intervention”[TIAB] OR “psychological interventions”[TIAB] OR “psychological treatment”[TIAB] OR “psychological treatments”[TIAB] OR "psychological crisis intervention"[TIAB] OR "psychological crisis interventions"[TIAB] OR “online support”[TIAB] OR “Hotlines”[Mesh] OR hotline[TIAB] OR tele-health[TIAB] OR “telemental health”[TIAB] OR debriefing[TIAB] OR “psychosocial support”[TIAB] OR “Cognitive Behavioral Therapy”[Mesh] OR “cognitive behavioural”[TIAB] OR “cognitive behavioral”[TIAB] OR “Eye Movement Desensitization Reprocessing”[Mesh] OR desensitization[TIAB] OR desensitisation[TIAB] OR EMDR[TIAB] OR psycho-education[TIAB] OR “Mindfulness”[Mesh] OR mindfulness[TIAB] OR “Meditation”[Mesh] OR meditation[TIAB] OR mentalization[TIAB] OR mentalisation[TIAB] OR “stress management”[TIAB] OR “stress monitoring”[TIAB] OR “resilience planning”[TIAB] OR coping[TIAB] OR "Mental Health"[Mesh] OR “mental health”[TIAB] OR “psychological impact”[TIAB] OR “Stress Disorders, Post-Traumatic”[Mesh] OR “Stress Disorders, Traumatic, Acute”[Mesh] OR PTSD[TIAB] OR "Stress, Psychological"[Mesh] OR Stress[TIAB] OR traumatization[TIAB] OR traumatisation[TIAB] OR “psychological problems”[TIAB] OR "Anxiety"[Mesh:NoExp] OR anxiety[TIAB] OR “Mental Fatigue”[Mesh] OR “mental fatigue”[TIAB] OR burnout[TIAB] OR burn-out[TIAB] OR "Depression"[Mesh] OR Depression[TIAB] OR "sleep problems"[TIAB] OR insomnia[TIAB] OR "Sleep Initiation and Maintenance Disorders"[Mesh:NoExp]

**Embase (via the Embase.com interface)**

‘healthcare professional’:ab,ti OR ‘healthcare professionals’:ab,ti OR ‘caregiver’/exp OR caregiver*:ab,ti OR (care NEXT/1 provider*):ab,ti OR ‘Physician’/exp OR physician*:ab,ti OR doctor*:ab,ti OR ‘Nurse’/exp OR nurse*:ab,ti OR ‘hospital personnel’/exp OR ‘paramedical personnel’/exp OR ‘clinical laboratory personnel’/exp OR ‘medical staff’/exp OR ‘medical staff’:ab,ti OR ‘medical workers’:ab,ti OR ‘health workers’:ab,ti OR ‘health professionals’:ab,ti OR ‘healthcare workers’:ab,ti OR ‘medical personnel’:ab,ti OR paramedic*:ab,ti OR volunteer*:ab,ti OR ‘general practitioner’/exp OR (general NEXT/1 practitioner*):ab,ti OR ‘first responder’:ab,ti OR ‘emergency medical technicians’:ab,ti

AND

‘epidemic’/exp OR epidemic*:ab,ti OR outbreak:ab,ti OR pandemic*:ab,ti OR ‘public health emergency’:ab,ti OR ‘SARS coronavirus’/exp OR SARS:ab,ti OR ‘Middle East respiratory syndrome coronavirus’/exp OR MERS:ab,ti OR ‘Ebolavirus’/exp OR ebola:ab,ti OR ‘Zika virus’/exp OR ‘Zika fever’/exp OR zika:ab,ti OR ‘Coronavirus infection’/exp OR ‘corona virus’:ab,ti OR ‘covid-19’:ab,ti

AND

‘mental health service’/de OR ‘counseling’/exp OR counseling:ab,ti OR ‘psychological assistance’:ab,ti OR (psychological NEXT/1 intervention*):ab,ti OR (psychological NEXT/1 treatment*):ab,ti OR ‘psychological crisis intervention’:ab,ti OR ‘psychological crisis interventions’:ab,ti OR ‘online support’:ab,ti OR ‘hotline’/exp OR hotline:ab,ti OR tele-health:ab,ti OR ‘telemental health’:ab,ti OR ‘debriefing’/exp OR debriefing:ab,ti OR ‘psychosocial support’:ab,ti OR ‘cognitive behavioral therapy’/exp OR ‘cognitive behavioural’:ab,ti OR ‘cognitive behavioral’:ab,ti OR 'eye movement desensitization and reprocessing'/exp OR desensitization:ab,ti OR desensitisation:ab,ti OR EMDR:ab,ti OR psycho-education:ab,ti OR ‘mindfulness’/exp OR mindfulness:ab,ti OR ‘meditation’/exp OR meditation:ab,ti OR mentalization:ab,ti OR mentalisation:ab,ti OR ‘stress management’:ab,ti OR ‘stress monitoring’:ab,ti OR ‘resilience planning’:ab,ti OR coping:ab,ti OR ‘mental health’/exp OR ‘psychological impact’:ab,ti OR ‘mental health’:ab,ti OR ‘posttraumatic stress disorder’/exp OR ‘acute stress disorder’/exp OR PTSD:ab,ti OR ‘mental stress’/exp OR stress:ab,ti OR traumatization:ab,ti OR traumatisation:ab,ti OR ‘psychological problems’:ab,ti OR ‘anxiety’/de OR anxiety:ab,ti OR 'dysthymia'/exp OR ‘mental fatigue’:ab,ti OR ‘caregiver burnout’/exp OR burnout:ab,ti OR burn-out:ab,ti OR ‘depression’/exp OR depression:ab,ti OR ‘sleep problems’:ab,ti OR insomnia:ab,ti OR ‘insomnia’/de

**PsycINFO (via the APA PsycNET database)**

It=(“Caregivers” OR “Physicians” OR “Nurses” OR “Medical Personnel” OR “Paramedics” OR “Allied Health Personnel” OR “General Practitioners”) OR ab=(“healthcare professional” OR “healthcare professionals” OR “caregiver” OR “care provider*” OR “physician*” OR “doctor*” OR “nurse*” OR “medical staff” OR “medical workers” OR “health workers” OR “health professionals” OR “healthcare workers” OR “medical personnel” OR “paramedic*” OR “volunteer*” OR “general practitioner*” OR “first responder” OR “emergency medical technicians”) OR ti=(“healthcare professional” OR “healthcare professionals” OR “caregiver” OR “care provider*” OR “physician*” OR “doctor*” OR “nurse*” OR “medical staff” OR “medical workers” OR “health workers” OR “health professionals” OR “healthcare workers” OR “medical personnel” OR “paramedic*” OR “volunteer*” OR “general practitioner*” OR “first responder” OR “emergency medical technicians”)

AND

It=(“Epidemics”) OR ab=(“epidemic*” OR “outbreak” OR “pandemic*” OR “public health emergency” OR “SARS” OR “MERS” OR “ebola” OR “zika” OR “corona virus” OR “covid-19”) OR ab=(“epidemic*” OR “outbreak” OR “pandemic*” OR “public health emergency” OR “SARS” OR “MERS” OR “ebola” OR “zika” OR “corona virus” OR “covid-19”) OR ti=(“epidemic*” OR “outbreak” OR “pandemic*” OR “public health emergency” OR “SARS” OR “MERS” OR “ebola” OR “zika” OR “corona virus” OR “covid-19”)

AND

It=(“Mental Health Services” OR “Counseling” OR “Hot Line Services” OR “Debriefing (psychological)” OR “Cognitive Behavior Therapy” OR “Eye Movement Desensitization Therapy” OR “Mindfulness” OR “Meditation” OR “Mental Health” OR “Posttraumatic Stress Disorder” OR “Acute Stress Disorder” OR “Psychological Stress” OR “Anxiety” OR “Fatigue” OR “Occupational Stress” OR “Depression (Emotion)” OR “Major Depression” OR “Insomnia”) OR ab=(“counseling” OR “psychological assistance” OR “psychological intervention*” OR “psychological treatment*” OR "psychological crisis intervention*" OR “online support” OR “hotline” OR “tele-health” OR “telemental health” OR “debriefing” OR “psychosocial support” OR “cognitive behavioural” OR “cognitive behavioral” OR “desensitization” OR “desensitisation” OR “EMDR” OR “psycho-education” OR “mindfulness” OR “meditation” OR “mentalization” OR “mentalisation” OR “stress management” OR “stress monitoring” OR “resilience planning” OR “coping” OR “psychological impact” OR “mental health” OR “PTSD” OR “stress” OR “traumatization” OR “traumatisation” OR “psychological problems” OR “anxiety” OR “mental fatigue” OR “burnout” OR “burn-out” OR “depression” OR “sleep problems” OR “insomnia”) OR ti=(“counseling” OR “psychological assistance” OR “psychological intervention*” OR “psychological treatment*” OR "psychological crisis intervention*" OR “online support” OR “hotline” OR “tele-health” OR “telemental health” OR “debriefing” OR “psychosocial support” OR “cognitive behavioural” OR “cognitive behavioral” OR “desensitization” OR “desensitisation” OR “EMDR” OR “psycho-education” OR “mindfulness” OR “meditation” OR “mentalization” OR “mentalisation” OR “stress management” OR “stress monitoring” OR “resilience planning” OR “coping” OR “psychological impact” OR “mental health” OR “PTSD” OR “stress” OR “traumatization” OR “traumatisation” OR “psychological problems” OR “anxiety” OR “mental fatigue” OR “burnout” OR “burn-out” OR “depression” OR “sleep problems” OR “insomnia”)
