# Supplementary material for: Factors affecting mental health of health care workers during coronavirus disease outbreaks (SARS, MERS & COVID-19): A rapid systematic review
Source: PLoS One. 2020 Dec 15;15(12):e0244052. doi: 10.1371/journal.pone.0244052 (PMC7737991; doi:10.1371/journal.pone.0244052)
Supplement: Attachment — Submitted filename: Reply to reviewers comments.docx [file pone.0244052.s006.docx]

**Reply to the remarks of the reviewers of manuscript ID PONE-D-20-11380**

Dear Dr. Miller

Dear Editor

Thank you for the work done on manuscript ID PONE-D-20-11380 entitled "Factors affecting mental health of health care workers during coronavirus disease outbreaks: a rapid systematic review" by Niels De Brier, Stijn Stroobants, Philippe Vandekerckhove and Emmy De Buck.

Reviewers commented positively on our submission and had some comments and suggestions. We now have revised the manuscript and believe that we have upgraded its quality. Below, we detail how we have addressed all of the individual points raised. We hope that the revisions made and responses given are clear such that the manuscript will now be acceptable for publication.

Yours sincerely,

Niels De Brier, corresponding author on behalf of all co-authors.

**Journal requirements**

Please ensure that your manuscript meets PLOS ONE's style requirements, including those for file naming

We doublechecked the journal guidelines and ensured that the manuscript meets the PLOS ONE's style requirements.

Thank you for stating the following in the Competing Interests section: "I have read the journal's policy and the authors of this manuscript have the following competing interests: the activities of the Belgian Red Cross include the provision of psychosocial first aid to laypeople."

Please confirm that this does not alter your adherence to all PLOS ONE policies on sharing data and materials, by including the following statement: "This does not alter our adherence to PLOS ONE policies on sharing data and materials.” Please include your updated Competing Interests statement in your cover letter; we will change the online submission form on your behalf.

In response, we updated the Competing Interest statement in the cover letter as follows: “*I have read the journal's policy and the authors of this manuscript have the following competing interests: the activities of the Belgian Red Cross include the provision of psychosocial first aid to laypeople. This does not alter our adherence to PLOS ONE policies on sharing data and materials*.”

**Reviewer #1**

Thank you for the opportunity to review this timely and well-written manuscript.

We thank the reviewer for his positive comment and for providing clear guidance on how to upgrade the quality of the manuscript.

Please state explicitly that the systematic review followed the steps outlined in Preferred Reporting Items for Systematic Reviews and Meta-Analyses (PRISMA).

In response, the sentence in lines 52-53 now reads as follows: “*The reporting of the systematic literature reviews was done according to the steps outlined in Preferred Reporting Items for Systematic Reviews and Meta-Analyses (PRISMA) (see S1 Table)*”.

Why was the project not registered with PROSPERO? I suspect due to time constraints and desire to publish the findings to aid with the pandemic, but please report the reason.

To address this comment, the sentence in lines 49-52 now reads as follows: “*Because of the timely relevance of the findings to support time-sensitive decisions during the COVID-19 pandemic, we decided to conduct a rapid systematic review. Due to time constraints inherent to the development of a rapid systematic review, no protocol for the systematic literature searches was registered beforehand with PROSPERO.*”

Was a librarian involved in developing the search strategies?

The search strategies were developed by review authors NDB and EDB who are trained information specialists and the sentence in lines 60-61 now read as follows: “*Two information specialists independently developed a search strategy based on search terms describing the HCW, the epidemic, and either mental health outcomes or interventions (full search strategies can be found in S2 Text)*.”

Were relevant bibliographies also searched?

No bibliographies were searched due to time constraints. We now acknowledge this limitation in the manuscript in line 243: “*Moreover, relevant bibliographies and clinical trial registries were not searched*.”

Were searches limited by date or publication status?

There were no constraints in selection criteria regarding publication date or status. In response, the sentence in lines 82-83 now reads as follows: “*Other: Only English language studies were included and there were no restrictions regarding publication date or status*.”

Were clinical trial registries also searched to limit publication bias, including: ClinicalTrials.gov, World Health Organization International Clinical Trials Registry Platform (WHO ICTRP), and the Australian New Zealand Clinical Trials Registry (ANZCTR), etc.?

No clinical trial registries were searched due to time constraints. We now acknowledge this limitation in the manuscript in line 243: “*Moreover, relevant bibliographies and clinical trial registries were not searched*.”

Inclusion / Exclusion criteria need to be more completely described.

To address this comment, we have now substantially revised the description of the inclusion and exclusion criteria in lines 65-83 as follows:

*“Studies were eligible if they addressed the PICO question and met the following inclusion and exclusion criteria:*

- *Population: Included: studies with HCWs in the (pre-)hospital setting during an outbreak of a coronavirus infection, causing the following diseases: SARS, MERS, COVID-19. Excluded: studies dealing with other infectious disease outbreaks (e.g. ebola and H1N1 virus).*
- *Risk or protective factors: Included: studies describing any risk factor or protective factor, which is relevant to take into account when developing either prevention programs or mental health interventions for HCWs. Excluded: studies describing non-modifiable factors such as gender, age and professional title* *and factors associated with impact on personal life. Relevant factors were discussed with content experts.*
- *Outcome: Included: any mental health outcome or psychological wellbeing.*
- *Study design: Included: (i) the studies of a systematic review if the search strategy and selection criteria were clearly described and if at least three electronic databases or relevant bibliographies were searched; (ii) experimental studies: (quasi or non-)randomized controlled trial (RCT), controlled before and after studies or controlled interrupted time series and (iii) observational studies: cohort and case-control studies, controlled before and after studies and controlled interrupted time series and cross-sectional studies, but measures should have controlled for confounding factors (e.g. matching, multivariate regression analyses). Excluded: cross-sectional studies which did not control for confounding factors, case series, letters, qualitative studies, conference abstracts and PhD theses.*
- *Other: Only English language studies were included and there were no restrictions regarding publication date or status.”*

The exact outcomes need to be more explicitly described. For example, it is not sufficient to state: Outcomes were categorized 67 in 8 categories, based on the measurement scales that were used. Risk and protective factors were categorized in 68 6 and 4 thematic categories, respectively. One should state the exact variables and scales used. Provide references for the validated scales.

We agree with the reviewer and have now described the outcomes more explicitly in the Results section as outlined below. The references of the exact outcome measures and scales used are provided in Table 1 and S3 and S4 Table.

Lines 103-110*: “The studies reported on a wide range of mental health outcomes which could be categorized in eight categories, based on the measurement scales that were used and detailed in Table 1: acute stress disorder/post-traumatic stress (mainly based on versions of the Impact of Event Scale), anxiety-related symptoms, depression-related symptoms, (perceived) stress, emotional exhaustion and burnout, sleep problems (including insomnia symptoms), anger and general symptoms of psychopathology including nonspecific outcomes mental distress, emotional distress, psychological distress, psychological disorder psychiatric morbidity, psychiatric symptoms, general mental health and negative emotional experience**. “*

Moreover, the sentence in lines 110-111 now reads as follows: “*The study characteristics, including exact outcome measures and scales used, are summarized in Table 1 and S3 Table.*”

Finally, we added numbers to Table 1 to transparently highlight the link between the outcome measure/scale used and the thematic category. The Table caption now reads as follows: “*Table 1. Study characteristics. Outcome measures sharing the same number in superscript belong to the same thematic category.*”

It is good that they used the GRADE system for grading evidence.

We thank the reviewer for his positive comment.

A visual representation of risk-of-bias assessments (e.g. RoB graph) would be helpful.

In response, we made a risk of bias graph as Fig. 2 and the caption reads as follows: ”Risk of bias graph showing each item presented as percentages across all observational studies with green: low risk of bias, yellow: unclear risk of bias, red: high risk of bias and grey: not applicable.

Please report risk of publication bias using the Egger or Begg-Mazumdar methods (or other validated method).

Based on the Cochrane and GRADE handbook, tests for funnel plot asymmetry should be used only when there are at least 10 individual studies included in the meta-analysis, because when there are fewer studies the power of the tests is too low to distinguish chance from real asymmetry. Besides the fact that we were not able to perform meta-analyses due to different effect sizes, there were less than 10 studies for each outcome per factor.

To address this comment and highlight this study limitation, the sentence in lines 245-246 now reads as follows: “*A formal assessment of publication bias was not possible due to large variation in outcome measures and effect sizes*.”

Please describe method used to determine heterogeneity, and what level of heterogeneity (e.g. I2 ≥50%, ≥75%, etc.) was the threshold beyond which data would not be suitable to combine into a meta-analysis.

As highlighted in lines 9-10, 88-89 and 331-333, a meta-analysis was not possible because of a high variety in risk factors, outcomes and effect measures (adjusted for different confounding factors) and we synthesized the data where possible using vote counting based on direction of effect by comparing the number of comparisons showing harm and benefit, regardless of the statistical significance or size of their results. As a consequence, we were not able to perform I^2^-statistics.

In response, we highlighted this limitation in lines 253-255: “*Third, since there was a high variation in outcome measures, risk factors, and measurement tools, it was difficult to synthesize the findings, and we used vote counting based on direction of effect as synthesis method.* *As a consequence, statistical heterogeneity could not be assessed.*”

Page 4, Line 80: please list subject # in parentheses behind “28 studies”

In response, the sentence in lines 99-100 now reads as follows: *“Out of 2605 references we selected 28 relevant studies (22523 participants) (Fig. 1), of which 27 cross-sectional studies, and one uncontrolled before-and-after study.”*

Results: when listing results for an aggregate number of studies (e.g. 4 included studies …) please list the (n) in parentheses.

This comment is readily taken care of.

Discussion: Many US physicians have self-imposed a form of quarantine from family (only going to work, living in garage, office, car, etc.) so as not to risk exposing them.

In response, we added this discussion to lines 314-315: “*Of note, attention should also be paid to HCWs who are self-quarantined to avoid infecting family members*.”

Is information available about the effects of healthcare workers being displaced from home / family.

Factors related to the personal life of the HCWs were not included in this review. To address this comment, the sentence in lines 71-72 reads as follows: *“Excluded:* *studies describing non-modifiable factors such as gender, age and professional title and factors associated with impact on personal life.”*

Line 269: Does the evidence suggest a most-effective mechanism for employers to check in / monitor the mental health of their front-line healthcare workers?

The evidence we gathered does not put forward a most effective monitoring strategy in the context of infectious disease outbreaks. We recommend monitoring of health care workers based upon their increased risk of developing mental health problems. Considering the significance of support factors, we would suggest that any form of monitoring is executed from a viewpoint of supportive concern (not a viewpoint of maladjustment detection), putatively promoting sense of support in HCWs. In response, we added the following sentence in line 293: “*Monitor from a viewpoint of supportive concern*.”

Limitations
- Not registered with PROSPERO

- Search strategy not developed by librarian

- Restricted only to English language

- Many important databases left out of search strategy (many of which could have been searched in English).

- Determination of inclusion/exclusion performed by 1 person.

- Data extraction performed by 1 person.

- Needs risk-of-bias graph.

- Risk of publication bias not reported.

- Heterogeneity not reported.

In response, we incorporated the individual points raised in the limitation section in the discussion in lines 240-261 as follows:

*“This systematic review has several limitations. First, because of time pressure of making timely relevant results available during the COVID-19 pandemic, no protocol was registered with PROSPERO, study selection and data extraction was done by only one reviewer and only English language studies were included. We only screened a limited number of databases. Moreover, relevant bibliographies and clinical trial registries were not searched. On the other hand, we used a sensitive search strategy, surveilled relevant grey literature, and two reviewers independently compared the included studies against the selection criteria. A formal assessment of publication bias was not possible due to large variation in outcome measures and effect sizes. Second, the quality of the included studies was very low, as the majority of studies had a cross-sectional design, which made it difficult to infer causal relationships from the results. However, within the available cross-sectional studies, we decided to only focus on those studies that controlled for confounding factors. Of note, the included studies did not involve the occupational mental health of HCWs prior to the disease outbreak as a control measure. Recent systematic reviews identified through an abbreviated literature search and screening clearly showed that especially occupational stress and burnout are already highly prevalent (even as high as 80%) among medical doctors and nurses in the pre-pandemic workplace [50-52]. Third, since there was a high variation in outcome measures, risk factors, and measurement tools, it was difficult to synthesize the findings, and we used vote counting based on direction of effect as synthesis method. As a consequence, statistical heterogeneity could not be assessed. To avoid fragmentizing the body of evidence, we combined several mental health outcomes in this analysis, for the different categories of risk/protective factors. Vote counting provides no information on magnitude of effect and takes no account of the differences in relative sizes of each study. Since the risk and protective factors were clustered in ten thematic categories, these categories (in)directly involve multiple affiliated factors and, hence, this synthesis method is not a nuanced approach. By way of example, witnessing multiple deaths is related to ‘level of disease exposure’ and risks of transmission to family present was categorized under ‘risk perception and fear’.”*

**Reviewer #2**

You are to be commended for performing the review in such a short time

We thank the reviewer for his/her positive comments.

Search: You did not mention languages searched. Which did you search?

In response, the sentence in lines 82-83 now reads as follows: “*Other: Only English language studies were included and there were no restrictions regarding publication date or status.*”

Results:

You identified 23 studies about SARS, 3 about MERS and 2 Chinese studies about Covid-19. The fact that there are only two Covid studies should appear in your title and be emphasised in your abstract and conclusions

We agree with the reviewer and added this information now to the abstract, the summary section of the discussion and conclusion as outlined below. Since we were already transparent about the characteristics of the included studies throughout the result section (lines 101-103, Table 1 and Table S3) and now emphasized this information also in the abstract, discussion and conclusion, we prefer to only highlight the selection criteria regarding population, i.e. coronavirus infections, in the title.

We added the following sentences to

lines 14-15: “*Most of these studies (n=23) were performed during the SARS outbreak, three during the MERS outbreak and two during the current COVID-19 pandemic.*“

lines 224-226: “*Most of the studies were performed during the SARS outbreak (n=23) while three studies during the MERS outbreak and two very recent studies during the current COVID-19 pandemic*.”

lines 232-234: “*We only included two studies on COVID-19 that fulfilled our selection criteria, which is probably due to our search date on March 24, which is still quite early in the pandemic*. *Other studies will probably come available the coming months.*”

And lines 336-337: “*Only two studies on COVID-19 that fulfilled the eligibility criteria were identified but the body of evidence is expected to grow the coming months*.”

You have identified these risk factors; direct contact, high risk units, high exposure risk, and working on the front line

You have identified these protective factors: clear communication, social support, sense of control, and coping ability, and less about physical safety and training.

In the media the health care workers and populations around the world have emphasised also the slowness of authorities to respond to the crisis, slow and inadequate social distancing and quarantining, the absence of personal protective equipment, witnessing multiple deaths, risks of transmission to family members, and long shifts and fatigue. There are thus some differences between these and the outcomes you found. Do you know an expert who has done media searches and has the software ready or could you put out a call who could identify the key words associated with Covid 19 to provide more evidence about Covid-19? and run a separate search including terms for health care workers.

We find it difficult to integrate this suggestion, because of the methodological rigourness of our systematic review. Although findings reported in the media could be timely relevant, they are mostly based on expert opinion, anecdotical evidence or case studies. These study types do not meet our predefined eligibility criteria, stating that studies should have taken measures to control for confounding factors. News reports extracted from peer-reviewed publications (which controlled for confounding factors) should be identified with the sensitive search strategy and included in this manuscript. Moreover, we already summarized the findings from the excluded studies on COVID-19 in the discussion on lines 234-239.

The risk factors highlighted by the reviewer are implicitly in line with factors identified in our review. We now elaborated on the scope of the identified risk factors as follows in lines 258-261: “*Since the risk and protective factors were clustered in ten thematic categories, these categories (in)directly involve multiple affiliated factors and, hence, this synthesis method is not a nuanced approach. By way of example, witnessing multiple deaths is related to ‘level of disease exposure’ and risks of transmission to family present was categorized under ‘risk perception and fear’*”.

Moreover, while the evidence on mental health and psychosocial needs of patients, family members, HCWs, … during the COVID-19 outbreak is rapidly emerging, it is more important to update the findings of this review when new evidence becomes available rather than including too low certainty evidence from the media. In response, the sentences in lines 232-234 now read as follows: “*We only included two studies on COVID-19 that fulfilled our selection criteria, which is probably due to our search date on March 24, which is still quite early in the pandemic. Other studies will probably come available the coming months.*”

And in lines 324-326: “*Lastly, the multiple uncertainties about the COVID-19 outbreak and the rapidly growing research make it necessary to provide the scientific community with high-quality and timely updates of the relevant evidence*.”

You are right to emphasise the very low study quality due to variation in risk factors, outcomes and measuring tools and that needs to be strongly emphasised in your abstract and conclusions.

In response, we added following sentences to the abstract and conclusion

in lines 9-10: “*Meta-analysis was not possible because of high variation in risk factors, outcomes and effect measures*.”

in lines 23-25: “*Low quality cross-sectional studies currently provide the best possible evidence, and further research is warranted to confirm causality*.”

And in lines 331-336: “*Due to the high variation in outcome measures, risk factors, and measurement tools, it was not possible to perform a meta-analysis but the data were synthesized by using vote counting based on direction of effect. Although it is difficult to infer causal relationships from the evidence, cross-sectional studies currently provide the best possible evidence for developing practical recommendations. High-quality controlled studies are needed for establishing casual relationships and identifying the most effective interventions.*”

Another key problem is that there are no baseline measures pre-pandemic for any workers and you need to stress that. A search for systematic reviews of healthcare workers stress and depression could provide you with a baseline.

We thank the reviewer for his comment and we agree that adequate pre-pandemic control groups are lacking in the included studies. To address this comment, we elaborated on mental health of HCW prior to the COVID-19 outbreak in lines 249-253: “*Of note, the included studies did not involve the occupational mental health of HCWs prior to the disease outbreak as a control measure. Recent systematic reviews identified through an abbreviated literature search and screening clearly showed that especially occupational stress and burnout are already highly prevalent (even as high as 80%) among medical doctors and nurses in the pre-pandemic workplace [50-52].*”

Moreover, the sentence in lines 318-319 now reads as follows: “*These could include proper control groups and longitudinal designs, with a follow-up measurement after the crisis period*.”

Finally, we identified one systematic review with our search strategies dealing with critical incidents such as infectious disease outbreaks and terroristic attacks on incidence of post-traumatic stress symptoms, anxiety, and depression in exposed versus non-exposed health professionals (baseline measure). The sentence in lines 263-267 now reads as follows: “*One review (search date 2009) dealt with the relation of the occurrence of work-related critical incidents and mental health of HCWs, and confirmed that treating victims of terror or disease outbreaks or, more general, treating patients in critical care treatments, had a small to medium impact on post-traumatic stress symptoms, anxiety, and depression in HCWs compared with a control, non-exposed group [53]*”.
