# Supplementary material for: Factors affecting mental health of health care workers during coronavirus disease outbreaks (SARS, MERS & COVID-19): A rapid systematic review
Source: PLoS One. 2020 Dec 15;15(12):e0244052. doi: 10.1371/journal.pone.0244052 (PMC7737991; doi:10.1371/journal.pone.0244052)
Supplement: Attachment — Submitted filename: Reply to reviewers comments.docx [file pone.0244052.s007.docx]

**Reply to the remarks of the reviewers of manuscript ID PONE-D-20-11380R1**

Dear Dr. Miller

Dear Editor

Thank you for the work done on manuscript ID PONE-D-20-11380R1 entitled "Factors affecting mental health of health care workers during coronavirus disease outbreaks: a rapid systematic review" by Niels De Brier, Stijn Stroobants, Philippe Vandekerckhove and Emmy De Buck.

Reviewers had additional comments and suggestions to improve the manuscript. We now have updated the study selection by including a specific COVID-19 resource, profoundly revised the manuscript and believe that we have upgraded its quality. Below, we detail how we have addressed all of the individual points raised. We hope that the revisions made and responses given are clear such that the manuscript will now be acceptable for publication.

Yours sincerely,

Niels De Brier, corresponding author on behalf of all co-authors.

**Reviewer #1**

Please indent the first line of new paragraphs.

In response, all first lines of new paragraphs in the manuscript are now indented.

Line 33, spelling: outbreaks

This comment is readily taken care of. The sentence in lines 31-33 now reads as follows: “*Similar yet less widespread coronavirus epidemics have occurred in the past, notably the severe acute respiratory syndrome (SARS) and Middle East respiratory syndrome (MERS) outbreaks [2,3].*”

The limitations of the search methodology resulted in a large number of missed studies -- this really undermines the message and solid writing. Cutting out new (and often Asian) literature is a major limitation. Much of the new literature is not on pubmed yet. Some are available as pre-prints. Also, many studies from regions that encountered the virus first (e.g. China) are published in other (non-PubMed) databases (e.g. China National Knowledge Infrastructure (CHKD-CNKI); information/Chinese Scientific Journals database (CSJD-VIP); LILACS, etc.). Even common databases like SCOPUS and Web of Science were not searched.

Within a few minutes of searching I was able to identify many missed articles. A partial list includes:

Guo J, Liao L, Wang B, et al. Psychological Effects of COVID-19 on Hospital Staff: A National Cross-Sectional Survey of China Mainland. SSRN Electron J 2020; published online March 24. DOI:10.2139/ssrn.3550050.
Huang JZ, Han MF, Luo TD, Ren AK, Zhou XP. [Mental health survey of 230 medical staff in a tertiary infectious disease hospital for COVID-19]. Zhonghua Lao Dong Wei Sheng Zhi Ye Bing Za Zhi 2020; 38: E001.
Liu C, Yang Y, Zhang XM, Xu X, Dou Q-L, Zhang W-W. The prevalence and influencing factors for anxiety in medical workers fighting COVID-19 in China: A cross-sectional survey. medRxiv 2020; : 2020.03.05.20032003.
Liu Z, Han B, Jiang R, et al. Mental Health Status of Doctors and Nurses During COVID-19 Epidemic in China. SSRN Electron J 2020; published online March 24. DOI:10.2139/ssrn.3551329.
Zhu Z, Xu S, Wang H, et al. COVID-19 in Wuhan: Immediate Psychological Impact on 5062 Health Workers. medRxiv 2020; : 2020.02.20.20025338.

Zhang C, Yang L, Liu S, et al. Survey of Insomnia and Related Social Psychological Factors Among Medical Staff Involved in the 2019 Novel Coronavirus Disease Outbreak. Front Psychiatry 2020; 11: 306.
Tan BYQ, Chew NWS, Lee GKH, et al. Psychological Impact of the COVID-19 Pandemic on Health Care Workers in Singapore. Ann Intern Med 2020. DOI:10.7326/M20-1083
Lu W, Wang H, Lin Y, Li L. Psychological status of medical workforce during the COVID-19 pandemic: A cross-sectional study. Psychiatry Res 2020; 288: 112936. PMID: 32276196
Du J, Dong L, Wang T, et al. Psychological symptoms among frontline healthcare workers during COVID-19 outbreak in Wuhan. Gen Hosp Psychiatry 2020; published online April. DOI:10.1016/j.genhosppsych.2020.03.011.
A survey of psychological status and sleep quality of nursing interns during the outbreak of COVID-19 infection. J South Med Univ. 2020;3:346-350.

Research on the Model of Psychological Support and Tutoring in New Coronarvirus Pneumonia. Healthcare Res Pract. 2020:2:6-10.

Psychological problems and intervention of first-line medical staff in the new coronavirus pneumonia isolation area. J Xinjiang Med Univ. 2020;4:386-390.

Study on the status quo and correlation between stress response and sympathetic fatigue of clinical first-line medical staff fighting new coronavirus pneumonia. Pract J Cardiovasc Pulm Dis. 2020;2:9-12.
We cannot agree more with the reviewer. Initially, we had only included two studies on COVID-19 which is mainly due to our search date on March 24 (which is still quite early in the pandemic) and also due to our strict eligibility criteria (excluding studies that did not control for confounding, or did not include modifiable risk factors). New studies concerning COVID-19 and mental health have become available the past two months, and most of the abovementioned studies were published after our search date (and thus not picked up by our original search).

To address this comment and identify recently published studies on COVID-19, we now have searched the NIPH systematic and living map on COVID-19 evidence (<https://www.nornesk.no/forskningskart/NIPH_mainMap.html>). In this resource, PubMed results are supplemented by regular updates with material retrieved by searches performed by organizations such as WHO and CDC, and we could specifically filter studies with health care workers and focusing on aspects including mental health. In this way we identified **five additional studies that met our eligibility criteria**. Two of these studies (Lu et al. and Zhang et al.) were also listed by the reviewer above. The majority of the other studies listed above were now also identified with the additional search, but were excluded because they did not fulfill our selection criteria, e.g. either the study was still in preprint phase and not peer reviewed (e.g. Guo et al., Liu et al., Zhu et al.), no risk/protective factors were measured or the factors measured were non-modifiable, such as demographic factors (e.g. Tan et al.), the study did not fulfill the study design criteria and/or no measures were taken to control for confounding (e.g. Du et al.) or the study was published in Chinese (e.g. Huang et al.).

More studies will become available the coming months and years and the rapidly growing research make it necessary to provide the scientific community with high-quality and timely updates of this rapid review. The ultimate goal of this rapid review is to timely provide a knowledge base for supporting the HCWs during the current acute outbreak but also to allow hospital-based managers and decision-makers to prepare prevention programs for a second wave or other infectious disease outbreaks in the future.

**In total, 33 observational studies were now selected with seven during the current COVID-19 pandemic** **Following study selection, the data of the new studies were extracted and tabulated (Table 1, Table S3-S5) and included in the data synthesis (Table 2). The new findings are also narratively synthesized in the results section. The results of Figures 1 and 2 are updated as well. All changes can be found in the marked-up copy of our manuscript.**

**Reviewer #2**

It is important that the title reflects that you analysed only two Covid 19 studies. Readers then know what to expect and can look elsewhere for more Covid studies in later publications or if you research an update.
We have identified five additional studies on COVID-19 that met our eligibility criteria trough a search in the NIPH living COVID-19 map. Since we now included seven very recently published studies on COVID-19, we believe it is not appropriate to emphasize the number of studies in the title but, in response to the reviewer’s comment and to be completely transparent, we now explicitly added the coronavirus diseases which are included in the review. The title now reads as: “*Factors affecting mental health of health care workers during coronavirus disease outbreaks (SARS, MERS & COVID-19): a rapid systematic review*”

You have no baseline measures. It is important from your literature search that you provide a baseline about stress levels of health care workers in non-pandemic situations.

We thank the reviewer for his comment and now further elaborated on non-pandemic mental health measures in HCWs, citing additional references (lines 261-271): “*Recent systematic reviews identified through an abbreviated literature search and screening clearly showed that occupational stress and burnout can be highly prevalent (even as high as 80%) among medical doctors and nurses in the pre-pandemic workplace [55-58]. Estimates are however complicated due to variability in definitions and assessment methods and differences between regions and occupational roles [55,58]. A cross-sectional study prior to the current pandemic in Spain showed that, although burnout is a major problem among nurses working in emergency departments, the average level of perceived stress was found within a normal range [59]. The study by McAlonan et al. [39] showed that the perceived stress levels in both high- and low-risk HCWs during the SARS outbreak were higher than the normative value of a US community sample. Furthermore, two (meta-analytic) prevalence studies performed during the current COVID-19 pandemic showed that levels of anxiety and depression of the HCWs during the outbreak in China were significantly higher than that of the HCWs during the non-outbreak period or the national norm [10,60].*“

Of note, the objective of the review was to identify any risk of protective factor which is relevant to take into account when developing either prevention programs or mental health interventions for HCWs and not to map and compare prevalence rates of mental health problems (and thus studies only reporting incidences of mental health problems were excluded).
