# Supplementary material for: Factors affecting mental health of health care workers during coronavirus disease outbreaks (SARS, MERS & COVID-19): A rapid systematic review
Source: PLoS One. 2020 Dec 15;15(12):e0244052. doi: 10.1371/journal.pone.0244052 (PMC7737991; doi:10.1371/journal.pone.0244052)
Supplement: Attachment — Submitted filename: Reply to reviewers comments.docx [file pone.0244052.s008.docx]

**Reply to the remarks of the reviewers of manuscript ID PONE-D-20-11380R2**

Dear Dr. Frey

Dear Editor

Thank you for the work done on manuscript ID PONE-D-20-11380R2 entitled "Factors affecting mental health of health care workers during coronavirus disease outbreaks (SARS, MERS & COVID-19): a rapid systematic review" by Niels De Brier, Stijn Stroobants, Philippe Vandekerckhove and Emmy De Buck.

Reviewers had additional comments and suggestions to improve the manuscript. We now have revised the manuscript and believe that we have upgraded its quality. Below, we detail how we have addressed all of the individual points raised. We hope that the revisions made and responses given are clear such that the manuscript will now be acceptable for publication.

Yours sincerely,

Niels De Brier, corresponding author on behalf of all co-authors.

**Reviewer #1**

Reviewer #1 had no further comments on the revised manuscript.

**Reviewer #2**

The topic has resulted in many studies and as the discussion between the reviewer and the authors shows there are important differences of opinion. My recommendation is that the authors review the new studies found by the reviewer and apply their criteria and be clear which can be included, and then I suggest that the editor adjudicates. This is then fair to readers and medical staff.

We profoundly elaborated on this comment in the previous round of revision. As recommended by Reviewer #1 previously, we updated the search on May 28 and we included five additional studies on COVID-19 that met our eligibility criteria. The initial search date was on March 24 which was still quite early in the pandemic and the newly found studies were published after our search date (and thus not picked up by our original search).

**Reviewer #3**

The authors in this systematic review attempted to identify the risk and protective factors for mental health outcomes in health care workers during coronavirus epidemics. The topic is important, pertinent and timely. However, I have some concerns about this study:

We thank the reviewer for highlighting the timely relevance of this rapid systematic review.

1- I appreciate short and concise introductions, particularly in meta-analysis, and to a lesser extent in systematic reviews. However, the current state of knowledge in the field is not sufficiently detailed. In order to identify the risk and protective factors for coronavirus-related mental health outcomes, those outcomes need to be characterized and described.

In response, we further detailed the emerging state of knowledge in the introduction and also included the description of the mental health outcomes in the introduction in lines 39-50 as follows: “*Moreover, high rates of mental health problems among physicians, nurses and hospital-based personnel during coronavirus disease outbreaks have been frequently described [9,10]. Mental health problems generally involve a constellation of changes in thinking, feeling and/or behavior, which are deemed undesirable by the person experiencing them and/or by his environment. Such changes can present within a broad range of severity from life’s daily hassles to diagnosable psychiatric disorders. Pappa et al. [11] and da Silva [12] provide early evidence that a high proportion of HCWs experience significant levels of anxiety, depression, stress and insomnia during the COVID-19 pandemic and Pan et al. [10] confirmed that the anxiety level of Chinese HCWs significantly increased during the outbreak of COVID-19. Another recent meta-analysis revealed that HCWs exposed to SARS/MERS/COVID-19 reported symptoms of e.g. fear, insomnia, psychological distress, burnout and anxiety [13]. Although these recent meta-analyses clearly indicate high pressure on the mental health of HCWs during the COVID-19 pandemic, personal, social and organizational factors associated with their vulnerability or resilience have not been synthesized.”*

2- PROSPERO is prioritizing COVID-related submissions and are registering protocols within days. The registration would have been recommendable, not only to avoid to a certain extent post-hoc decisions, but because they provide useful recommendations. In this case, I believe they would have asked the authors to better describe the PICO framework.

To scientifically support the Belgian Red Cross’ response to the early stages of the COVID-19 pandemic, the review was developed in a very short time frame between March 24 and April 7 with an update on May 28. Due to the given time constraints, no protocol was published beforehand. We acknowledged the limitation of this decision in lines 285-287 as follows: “*This systematic review has several limitations. First, because of time pressure of making timely relevant results available during the COVID-19 pandemic, no protocol was registered with PROSPERO, study selection and data extraction was done by only one reviewer and only English language studies were included*.”

To better describe the PICO framework, thereby taking into account the comments outlined below, we further elaborated on the eligibility criteria as follows in lines 80-109:

- *Population: Included: studies targeted at all staff which are/were active within a health care setting (e.g. a whole hospital or specific unit, health center, or community health network) during an outbreak of a coronavirus infection, causing the following diseases: SARS, MERS, COVID-19. Excluded: studies dealing with other infectious disease outbreaks (e.g. ebola and H1N1 virus).*
- *Risk or protective factors: Included: studies describing any modifiable risk factor or protective factor,* *which is relevant to take into account when developing either prevention programs or mental health interventions for HCWs in the context of an infectious disease outbreak. Risk factors are here defined as characteristics at organizational, social and personal level that putatively precede and are associated with poor mental health outcomes in HCWs. Protective factors are positive influences that may protect HCWs for developing mental health problems during coronavirus disease outbreaks. Modifiable factors include behaviors, experiences and exposures that may be controlled and changed for maximizing resilience of HCWs during and after these crises. Examples of modifiable factors are: direct contact with patients, dissatisfaction with procedures, changes in work demands, being quarantined as HCW, fear of infection, stigma, vulnerability, clear communication of directives, professional support, social support, perceived self-efficacy, sufficient precautionary measures, training in protection… We adhered to the author’s interpretation on the classification of risk/protective factors and outcome measures. Excluded: studies describing non-modifiable factors such as gender, age, family history and professional title and factors associated with impact on personal life. Relevant factors were discussed with content experts.* *Studies solely dealing with prevalence or incidence rates of mental health outcomes are excluded since no data on risk or protective factors can be extracted.*
- *Outcome: Included: any mental health outcome or psychological wellbeing. Mental health problems can present within a broad range of severity. Hence, we not only focused on mental health outcomes reflecting psychiatric symptoms and/or caseness, but also included studies assessing mental health symptomatology reflecting common emotional and social undesirable changes in thinking, feeling and/or behavior. Mental health outcomes included physical symptoms such as pain and fatigue in as far as they were part of mental health questionnaires and thus considered putatively related to psychological suffering. Mental health outcomes were considered in the immediate, short- and long-term as long as association with risk/protective factors directly relevant to the disease outbreak could be examined. Excluded: physical health problems related to the inflammatory responses of the human body such as fever, cough, myalgias, chills, headaches, dyspnea, sore throat, nausea/vomiting and diarrhoea.*

3- The literature search is inefficient and under my point of view probably the main reason why relevant hits were lost along the process. Not sure the addition of keywords as “Zika virus’/exp”, “‘Zika fever’/exp”, “hotline:ab,ti” led to the inclusion of many relevant hits. However, demanding studies to include one of the keywords in the third paragraph in the literature search in order to be included, must have limited the detection capacity of relevant hits.

We agree with the reviewer that search terms related to the Zika and Ebola virus might be too broad regarding the PICO question but by including these terms we initially ensured that no studies with related infectious disease outbreaks were missed. Since there was a large body of evidence on coronavirus disease outbreaks, we decided to exclude studies dealing with Ebola or Zika viruses as a source of indirect evidence. Including the above-mentioned terms in the search strategy (using the Boolean ‘OR’ operator) made the search strategy more sensitive (broader) and not more specific, which could not have led to missing relevant records.

The third paragraph of the search strategy included either mental health outcomes or interventions. By combining multiple relevant and sensitive terms such as, amongst others, “mental health”, “psychosocial support” or “stress” all relevant records should be identified in the databases used. The Cochrane Handbook (Chapter 4) also demands that a set of terms dealing with the interventions is included in the search strategy. Searching for outcomes is optional but the outcome terms are here combined with the Boolean ‘OR’ operator with the intervention terms to increase sensitivity. Based on discussions with a content expert we included terms related to “hotline” in the search strategy since it is a potentially relevant psychological service which is often frequented by people in mental distress for providing relief. Furthermore, there were early reports of such services being specifically set up to support HCWs during the COVID-19 pandemic (e.g., Kang et al., 2020^1^).

The search strategies were co-developed by review authors NDB and EDB who are trained medical/healthcare information specialists and content experts (SB and KVP). By doing so, we ensured that we used a sensitive search strategy for detecting relevant studies. In addition to running these searches in the different databases, we also screened the COVID-19 living evidence map from the Nordic Institute of Public Health, containing all experimental and observational studies conducted in the context of COVID-19. This strategy allowed us to be very complete, and not to miss relevant studies. If certain studies were not included, the reason for this were our eligibility criteria or the search date.

^1^*Kang L, Li Y, Hu S, Chen M, Yang C, Yang BX, Wang Y, Hu J, Lai J, Ma X, Chen J, Guan L, Wang G, Ma H, Liu Z. The mental health of medical workers in Wuhan, China dealing with the 2019 novel coronavirus. Lancet Psychiatry. 2020, 7(3):e14.*

4- I am not convinced by the definition of population regarding exposure to coronavirus. Several studies evaluated HCW, particularly during SARS pandemic, both during outbreak and after the outbreak. It seem like authors considered posttraumatic stress symptoms as well, which may appear months/years after. Also, how was hospital setting defined? Were GPs or HCW working in the community excluded?

We indeed included studies assessing the mental health of health care workers (HCWs) which are/were active during an outbreak of a coronavirus infection (see S5 Table for assessment of recall bias). Furthermore, we included HCWs in all different settings. Mental health outcomes were considered in the immediate, short- and long-term as long as association with risk/protective factors directly relevant to the disease outbreak could be examined (see response to comment 6). Lastly, the risk factor of ‘level of disease exposure’ included, amongst others, direct contact with patients, working in high risk units, high risk of exposure, and working on the frontline (see lines 155-156).

In response, we now have better defined the population’s inclusion criteria in lines 80-82 as follows: “*studies targeted at all staff which are/were active within a health care setting (e.g. a whole hospital or specific unit, health center, or community health network) during an outbreak of a coronavirus infection, causing the following diseases: SARS, MERS, COVID-19.”*

5- The definition of a risk or a protective factor is really ambiguous and this is particularly problematic. This way it is not possible to replicate what the authors have done. For instance, looking at the studies in the following systematic review (PMID: 32658823) that were not included in this systematic review, it is difficult to know if it was an strict definition of risk/protective factor (or outcome), an inefficient literature search, or losses of studies during the screening process, which led to the authors missing studies that seem relevant to me.

We followed the author’s interpretation on the classification of risk/protective factors and outcome measures. In response, we now defined protective and risk factors as follows in lines 84-99: “*Risk or protective factors: Included: studies describing any modifiable risk factor or protective factor, which is relevant to take into account when developing either prevention programs or mental health interventions for HCWs in the context of an infectious disease outbreak. Risk factors are here defined as characteristics at organizational, social and personal level that putatively precede and are associated with poor mental health outcomes in HCWs. Protective factors are positive influences that may protect HCWs for developing mental health problems during coronavirus disease outbreaks. Modifiable factors include behaviors, experiences and exposures that may be controlled and changed for maximizing resilience of HCWs during and after these crises*. *Examples of modifiable factors are: direct contact with patients, dissatisfaction with procedures, changes in work demands, being quarantined as HCW, fear of infection, stigma, vulnerability, clear communication of directives, professional support, social support, perceived self-efficacy, sufficient precautionary measures, training in protection…* *We adhered to the author’s interpretation on the classification of risk/protective factors and outcome measures. Excluded: studies describing non-modifiable factors such as gender, age, family history and professional title and factors associated with impact on personal life. Relevant factors were discussed with content experts.*”

We carefully read the systematic review highlighted by the reviewer. While this review appraised the burden (type and frequency) of physical and mental health outcomes in the current literature, we complementary focused on the risk and protective factors which might be associated with mental health outcomes in HCWs. As a result, studies solely reporting prevalence figures were not included in our rapid systematic review. The exclusion criteria in lines 197-99 now read as follows: “*Studies solely dealing with prevalence or incidence rates of mental health outcomes are excluded since no data on risk or protective factors can be extracted*.” Moreover, we elaborated on the findings of this review in the introduction section in lines 47-48 as follows: “*Another recent meta-analysis revealed that HCWs exposed to SARS/MERS/COVID-19 reported symptoms of e.g. fear, insomnia, psychological distress, burnout and anxiety [13]”* Moreover, we added the following sentences to the discussion section in lines 325-327: “*Very recent meta-analyses confirmed that HCWs exposed to (SARS, MERS or) COVID-19 suffered from a wide range of mental health problems but no risk or protective factors were identified [10-13].”*

6- Further explanation of which outcomes were included and excluded would be advisable. How were physical and mental health outcomes defined and differentiated?

We agree with the reviewer that further explanation is advisable. However, as mental health takes into account physical symptoms without identifiable physical cause, physical and mental health symptoms cannot be entirely differentiated. However, we tried to differentiate these outcomes from physical health as relevantly as possible through focusing on mental health outcome questionnaires. Such questionnaires often include physical symptoms such as pain or fatigue as well (e.g., anxiety, burnout or PTSD-related scales), although always putatively related to psychological suffering.

To address this comment, the outcome’s inclusion and exclusion criteria now read in lines 100-109 as follows: “*Outcome: Included: any mental health outcome or psychological wellbeing. Mental health problems can present within a broad range of severity. Hence, we not only focused on mental health outcomes reflecting psychiatric symptoms and/or caseness, but also included studies assessing mental health symptomatology reflecting common emotional and social undesirable changes in thinking, feeling and/or behavior. Mental health outcomes included physical symptoms such as pain and fatigue in as far as they were part of mental health questionnaires and thus considered putatively related to psychological suffering. Mental health outcomes were considered in the immediate, short- and long-term as long as association with risk/protective factors directly relevant to the disease outbreak could be examined. Excluded: Physical health problems related to the inflammatory responses of the human body such as fever, cough, myalgias, chills, headaches, dyspnea, sore throat, nausea/vomiting and diarrhoea.”*

7- Pooling together any risk/protective factor with any mental health outcome to provide quantitative results seems questionable to me. Also, do not understand why the outcomes are too heterogenous for a meta-analysis, but not for vote counting.

According to the Cochrane Handbook (Chapter 12), legitimate reasons why it may not be possible to undertake a meta-analysis include amongst others different effect measures used across studies and methodological diversity. In our case, studies reported mean difference, adjusted odds ratio, or regression coefficient as effect measure and it was not possible to pool these different measures. Moreover, the risk/protective factors were evaluated with different scales and it is, hence, not legitimate to perform meta-analyses. Vote counting is based on the direction of effect and can be used according to the Cochrane Handbook when there is inconsistency in the effect measures or data reported across studies, as is the case for this rapid review. Vote counting only addresses the question: Is there any evidence of effect? It does not provide any information on the magnitude of effects and does not account for differences in the relative sizes of the studies. This decision was motivated in the abstract in lines 9-10: “*Meta-analysis was not possible because of high variation in risk factors, outcomes and effect measures*” and in the method section in lines 124-127: “*Since meta-analysis was not possible because of a high variety in factors, outcomes and effect measures (adjusted for different confounding factors), we synthesized the data where possible using vote counting based on direction of effect by comparing the number of comparisons showing harm and benefit, regardless of the statistical significance or size of their results [21]*.”

We now also further acknowledged the underlying limitations of this method in the discussion section in lines 308-318 as follows: “*Third, since there was a high variation in effect measures, risk factors, and measurement tools, it was difficult to synthesize the findings, and we used vote counting based on direction of effect as synthesis method. As a consequence, statistical heterogeneity could not be assessed. To avoid fragmentizing the body of evidence, we combined several mental health outcomes in this analysis, for the different categories of risk/protective factors. Vote counting provides no information on magnitude of effect and takes no account of the differences in relative sizes of each study. Since the mental health outcomes were grouped and the risk and protective factors were clustered in ten thematic categories, this synthesis method is hence not a nuanced approach.* *Indeed, these thematic categories (in)directly involve multiple affiliated factors and, by way of example, witnessing multiple deaths could be related to ‘level of disease exposure’ and risk of transmission to family was presently categorized under ‘risk perception and fear’.* *Nonetheless the results of the individual studies were tabulated and extensively discussed*”.

In addition to this way of summarizing the data, we also tabulated the results and quality of the individual studies in the supporting information (S4 Table and S5 Table), as well as extensively elaborated on these results in the result section, in order to also transparently report and present individual results to the reader.

8- I do not agree with the decision of considering p-value < 0.10 significant regardless of the method and this decision should be better justified.

To address this comment, we decided to consider p-value < 0.05 significant for the results of vote counting. The sentence in line 128 now reads as follows: “*A p-value < 0.05 was considered statistically significant.*” The discussion of the results on being quarantined as HCW were rewritten accordingly.

9- Including only studies in English in this topic is a limitation as many studies come from Asia.

We agree with the reviewer that this is a limitation, which we now acknowledged in the Discussion section. However, 80% of our included studies were conducted in Asia, which we now also mentioned in the Discussion section in lines 285-289 as follows: “*First, because of time pressure of making timely relevant results available during the COVID-19 pandemic, no protocol was registered with PROSPERO, study selection and data extraction was done by only one reviewer and only English language studies were included. The latter has resulted in the exclusion of some studies, particularly from Asian countries. However, we were able to detect 33 individual studies against the predefined eligibility criteria of which about 80% were conducted in Asia.*”
