# Supplementary material for: Factors affecting mental health of health care workers during coronavirus disease outbreaks (SARS, MERS & COVID-19): A rapid systematic review
Source: PLoS One. 2020 Dec 15;15(12):e0244052. doi: 10.1371/journal.pone.0244052 (PMC7737991; doi:10.1371/journal.pone.0244052)
Supplement: Attachment — Submitted filename: Reply to reviewers comments.docx [file pone.0244052.s009.docx]

**Reply to the remarks of the reviewers of manuscript ID PONE-D-20-11380R3**

Dear Dr. Frey

Dear Editor

Thank you for the work done on manuscript ID PONE-D-20-11380R3 entitled "Factors affecting mental health of health care workers during coronavirus disease outbreaks (SARS, MERS & COVID-19): a rapid systematic review" by Niels De Brier, Stijn Stroobants, Philippe Vandekerckhove and Emmy De Buck.

Reviewers commented positively on our manuscript and clearly stated that all comments have been addressed. Nevertheless, reviewer 2 had one final suggestion to improve the manuscript. Below, we detail how we have addressed this final point raised.

We have revised the manuscript four times profoundly, including two updates of our searches, and hope that the manuscript will now be acceptable for publication given the timely relevance of the review’s conclusions.

Yours sincerely,

Niels De Brier, corresponding author on behalf of all co-authors.

**Reviewer #1**

Reviewer #1 had no further comments on the revised manuscript.

**Reviewer #2**

I have read the detailed responses to the comments of reviewer#3 and you have endeavoured to reply to all of them in great detail.

We thank the reviewer for his positive comment.

The problem of having an up to date review especially when there is a time sensitive outcome (as with your review, where health care workers and administrators want to know your findings and apply them) is difficult.

Your last search was May 28 in a Covid-19 database. Nearly five months (June, July, August, September and now 22 October) have gone by. It would be reasonable for you to run your search strategy for studies after May 28 and simply state if you found any studies at low risk of bias and if they might change your conclusions substantially. If no such studies are found then you can say so. It is likely that studies on COVID-19 will have continued to be published after May 28 but less likely on the other two corona virus epidemics.

In response, we reran our search in the NIPH living COVID-19 map (PubMed results supplemented by regular updates with material retrieved by searches performed by organizations such as World Health Organization, WHO and Centers for Disease Control and Prevention, CDC) on October 26 and screened for additional relevant experimental or observational studies. We were not able to identify studies of higher quality than the studies already included, and provided this information in the discussion section. Moreover, to go beyond the comment of the reviewer, we also synthesized the results of the eight newly identified cross-sectional studies meeting the eligibility criteria. It is important to note that these low-quality studies did not reveal any new risk factors. Taken together, we now have added the following paragraph to the discussion section in lines 382-401:

“*Lastly, the multiple uncertainties about the COVID-19 outbreak and the rapidly growing research make it necessary to provide the scientific community with high-quality and timely updates of the relevant evidence. As this review deals with time sensitive results, we screened for additional relevant experimental or observational studies in the NIPH living COVID-19 map during the publication process (search date Oct 26). In this time period, no high-quality studies at low risk of bias which could substantially impact on the review’s conclusions were identified. Eight additional cross-sectional studies, which suffer from coexisting methodological issues, fulfilled the eligibility criteria. These studies did not reveal any new risk factors and further underpin the findings of this review. When briefly discussing the risk factors, studies by Cai et al. [63] and Wankowicz et al. [64] confirmed that the rate of mental health problems is significantly increased in frontline HCWs. Further, there is growing evidence that risk perception and health fear are independent factors for developing mental distress [65,66] while Sarboozi Hoseinabadi et al. [67] could not demonstrate a significant association between fear of COVID-19 infection and burnout symptomatology. It was also confirmed that loneliness because of social isolation as well as job stress are associated with increased mental distress or burn-out symptomatology, respectively [66,67]. Regarding the protective factors, further evidence shows that receiving adequate information and availability of protective measures are significantly associated with reduced severity of mental health problems [66,68,69]. Sarboozi Hoseinabadi et al. [67] could not demonstrate a significant association between social support from family and friends or hospital resources and COVID-19-related burnout. Finally, Huang et al. [70] found that problem-coping style is the common influencing factor for anxiety in nurses. It is of note that the studies of Arpacioglu et al. [65] and García-Fernández et al. [68] included HCWs as well as non-HCWs and results between groups could not be distinguished*.”

**Reviewer #3**

Thank you for your detailed response to my previous comments.

In my opinion the manuscript has improved a lot.

We thank the reviewer for his/her positive comment.
